# Comprehensive Proteomics Reveals Acute Osmoregulatory Responses in the Intestine of *Oreochromis mossambicus* Under Salinity, Alkalinity, and Combined Saline–Alkaline Stress

**DOI:** 10.3390/ani16193069

**Published:** 2026-09-29

**Authors:** Huanhuan Su, Dongmei Ma, Jiajia Fan, Zaixuan Zhong, Huaping Zhu

**Affiliations:** 1 Key Laboratory of Tropical & Subtropical Fishery Resource Application & Cultivation, Ministry of Agriculture and Rural Affairs, Pearl River Fisheries Research Institute, Chinese Academy of Fishery Sciences, Guangzhou 510380, China; 2 Heyuan Branch, Guangdong Laboratory for Lingnan Modern Agriculture, Heyuan 517000, China

**Keywords:** antioxidant enzymes, aquaculture, euryhaline fish, oxidative stress, saline–alkaline stress

## Abstract

Osmotic stress induces pleiotropic physiological disruptions in aquatic organisms; however, the intestinal proteomic responses of euryhaline teleosts to salinity, alkalinity, and saline–alkalinity exposure remain insufficiently characterized. In the present study, intestinal histopathology, antioxidant enzymatic activities, and proteomic profiles were investigated in *Oreochromis mossambicus* (Peters, 1852) subjected to 24-h acute individual and concurrent salinity-alkalinity challenges. Pronounced histopathological lesions and significant alterations in enzymatic antioxidant defenses were observed, as reflected by elevated glutathione peroxidase (GSH-Px) and malondialdehyde (MDA) levels alongside diminished catalase (CAT) activity. Proteomic analysis identified multiple differentially abundant proteins implicated in osmoregulation across all treatment groups, and subsequent pathway enrichment analysis indicated that stress exposure perturbed oxidative phosphorylation and immune-related signaling cascades, particularly under alkalinity and combined conditions. Collectively, this work provides the first comparative proteomic characterization of intestinal acute stress responses to osmotic stress in *O. mossambicus*, thereby enhancing our understanding of the molecular regulatory networks governing osmoregulation in euryhaline fish confronted with multifactorial environmental fluctuations.

## Simple Summary

Fish are often farmed in water that is saltier or more alkaline than freshwater, and they must cope with these changes quickly. We looked at what happened in the intestine (the gut) of Mozambique tilapia during the first 24 h in salty water, alkaline water, and a mixture of both. The gut showed tissue damage under the microscope, changes in natural defense molecules, and changes in the amounts of thousands of proteins, especially those that produce energy and those that control immunity. These results show how fish begin to respond to difficult water conditions within one day. This knowledge can help fish farmers and scientists protect fish health and improve farming in salty or alkaline waters.

## 1. Introduction

In response to the growing demand for safe and reliable animal protein, the aquaculture sector plays a vital role in delivering healthy and sustainable farmed products [1]. Owing to its notable tolerance to stressors arising from typical environmental conditions in aquaculture settings [2], tilapia has become a primary candidate for intensive farming systems, effectively meeting the expectations of both consumers and producers [3]. Although tilapia is taxonomically considered a freshwater species [4], its capacity to withstand brackish-water environments [5], renders it particularly valuable for aquaculture in regions where freshwater resources are scarce. Among the tilapia group, Mozambique tilapia (*Oreochromis mossambicus* Peters, 1852) is indigenous to estuarine habitats and the lower river courses extending from the Zambezi River to the southeastern coast of South Africa, and it is typically observed within approximately one mile of tidal influence. As a highly euryhaline species, *O. mossambicus* thrives across a broad salinity gradient, with tolerance levels exceeding 70‰ [6,7]. Given its distinctive physiological attributes, *O. mossambicus* has emerged as an excellent candidate for brackish-water aquaculture and holds promise as a model organism for environmental research; nevertheless, studies in this context remain comparatively sparse [8].

Given the progressive depletion of accessible freshwater resources and the rising consumer demand for high-quality aquatic protein, a growing body of research has directed attention toward the farming of euryhaline fish species in underutilized inland saline–alkaline terrains and nearshore brackish waters [9,10]. Salinity constitutes a critical environmental determinant [11], that induces alterations in osmotic pressure and consequently influences physiological homeostasis [12]. Prior investigations have predominantly concentrated on examining the effects of salinity on growth performance [13], body composition [14], plasma osmolality [15], antioxidant capacity [16], and metabolism and nutrient requirements [17]. However, the molecular mechanism of salinity adaptation of halophilic fishes has not been investigated in depth. Saline–alkaline water contains carbonate, which serves as a crucial buffer mechanism for preserving a steady pH. However, in aquatic habitats with minimal alkalinity, pH has been demonstrated to fluctuate. High alkaline pH exposure can block Na^+^ inflow in fish, resulting in an imbalance of osmotic pressure inside and outside the body, which can impede development or possibly result in death [18]. Water-quality conditions, including alkalinity and mineral composition, can influence the physiological performance and rearing environment of tilapia [19]. As revealed by the analysis of alkalinity transcriptome data, when exposed to a range of carbonate alkalinity concentrations, Nile tilapia (*Oreochromis niloticus*) underwent a variety of physiological alterations [20]. Fish engage in metabolic processes to maintain homeostasis under weak carbonate alkalinity conditions that are not favorable for fish survival [21,22,23]. However, this reaction has a detrimental effect on the excretion of nitrogen waste, which can affect the ability of fish to survive in extremely alkaline environments.

Environmental variables, including temperature, salinity, and dissolved oxygen, are known to modulate the cellular oxidative state and the generation of reactive oxygen species (ROS). Such factors can elicit the activation of antioxidant defense systems across various organs, thereby counteracting and neutralizing ROS [24]. Salinity exposure can disrupt physiological homeostasis in tilapia and induce osmotic and oxidative stress, leading to alterations in antioxidant defense mechanisms and tissue integrity [25]. Among the enzymatic antioxidants involved in ROS scavenging, superoxide dismutase (SOD), catalase (CAT), and glutathione peroxidase (GSH-Px) represent pivotal components of the antioxidant repertoire in fish [26]. Malondialdehyde (MDA), a byproduct of lipid peroxidation, can induce cross-linking of membrane proteins, elevate membrane permeability, and ultimately result in cellular injury [27,28]. Accordingly, MDA levels serve as a reliable indicator of the degree of lipid peroxidation occurring in vivo. Furthermore, the intestine, being in direct contact with external aquatic environments, is susceptible to morphological changes in response to physicochemical challenges [29]. In most monogastric vertebrates, including teleost fish, the intestinal epithelium constitutes the primary defensive barrier against deleterious agents within the gut lumen. Epithelial surfaces function as mechanical barriers that separate internal tissue compartments from the external milieu [30]. The intestinal mucosa and its epithelium are of critical importance, constituting the frontline defense against pathogenic agents in the gut lumen and playing a recognized role in both innate and adaptive immune responses [31]. Nevertheless, the molecular mechanisms underlying intestinal osmoregulation in euryhaline fishes under varying osmotic pressures—encompassing genes, proteins, and associated signaling pathways—remain largely unresolved [27].

Omics technologies hold considerable promise for elucidating the mechanisms by which environmental factors induce adverse effects in organisms [32,33]. These approaches are equally valuable for the identification of suitable biomarkers that reflect such effects [34,35]. Currently, proteomics ranks as the second most extensively employed omics platform, enabling systematic qualitative and quantitative profiling of the entire proteome in cells and tissues, and facilitating the identification of differentially expressed proteins (DEPs) as candidate biomarkers. Indeed, proteomic approaches have been successfully applied to characterize protein alterations in response to diverse environmental stressors, including harmful algal blooms, biocides, and salinity stress [36,37]. Although several studies have employed proteomics to investigate the effects and molecular mechanisms of osmoregulation under salinity stress in aquatic organisms [38], to the best of our knowledge, no comparative proteomic analyses have been reported that simultaneously examine organismal responses to salinity, alkalinity, and combined saline–alkaline stress. Therefore, further proteomic investigations are warranted to elucidate the molecular mechanisms elicited by salinity, alkalinity, and combined saline–alkaline stress.

A recent TMT-based proteomic study demonstrated that salinity, alkalinity, and combined saline–alkaline stress induce distinct proteomic responses in the gills of *O. mossambicus*, particularly involving ion transport, energy metabolism, immunity, and apoptosis [39]; however, whether similar molecular responses occur in the intestine under the same environmental conditions remains insufficiently characterized. Despite extensive research on intestinal ion and water transport mechanisms, significant knowledge gaps persist. To address these outstanding questions, we employed a quantitative proteomic strategy based on isobaric tandem mass tags (TMT) to profile the intestinal proteome of *O. mossambicus* acclimated to four experimental conditions: freshwater control, salinity stress, alkalinity stress, and combined saline–alkalinity stress. In parallel, we assessed the activities of key antioxidant enzymes (SOD, CAT, and GSH-Px) as well as malondialdehyde (MDA) levels, and performed histopathological examinations, in order to comprehensively compare intestinal responses across these distinct osmotic challenges. Notably, the present work complements our previous metabolomic study of the intestine of *O. mossambicus* exposed to the same stressors [40], in which metabolic shifts could not be assigned to specific molecular executors; by providing the corresponding protein-level landscape, the present study links the observed metabolic rearrangements to their putative protein regulators.

## 2. Materials and Methods

### 2.1. Ethics Approval

All animal experiments were reviewed and approved by the Animal Care and Use Committee of the Pearl River Fisheries Research Institute, Chinese Academy of Fishery Sciences. The ethical code was LAEC-PRFRI-2022-05-03, and the approval date was 19 May 2022. The 3-aminobenzoic acid ethyl ester (MS-222; Aladdin, Tokyo, Japan) with a concentration of 30 mg/L was used to anaesthetize *O. mossambicus* tilapia.

### 2.2. Animals and Treatment

A total of 1200 healthy *O. mossambicus* with a mean initial body weight of 26.57 ± 6.27 g were obtained from the Pearl River Fisheries Research Institute, Chinese Academy of Fishery Sciences (Guangzhou, China). Following a two-week acclimation period in freshwater (pH 7.8, temperature 28 °C), the fish were fed a commercial diet twice daily (at 09:00 and 17:00) at a daily ration of 5% of their body weight. All healthy fish were randomly assigned, using a random number table, to four experimental groups: a control group (C_C, freshwater), a salinity stress group (S_C, salinity 25 g/L), an alkalinity stress group (A_C, alkalinity 4 g/L), and a combined saline–alkalinity stress group (SA_C, salinity 25 g/L plus alkalinity 4 g/L) [39,40]. Each group comprised three replicate tanks, each stocked with 100 individuals and containing 140 L of water. Saline, alkaline, and combined saline–alkaline waters were prepared by dissolving commercial sea salt and sodium bicarbonate (NaHCO_3_) in dechlorinated, aerated freshwater to reach the nominal salinity (25 g/L) and alkalinity (4 g/L) of the respective treatments. All treatment waters were prepared 24 h before use and were continuously aerated. Water quality parameters, including temperature, pH, dissolved oxygen, salinity, and total alkalinity, were monitored during the experiment, and the measured values for each group are summarized as follows: temperature = 26 ± 2 °C; pH = 8.0 ± 0.2; dissolved oxygen = 6.0 ± 0.3 mg/L; salinity = 24 ± 0.2 g/L. The actual alkalinity of the water was determined by acid–base titration using 1 mol/L hydrochloric acid (HCl) with methyl orange as the indicator; the measured alkalinity was approximately 4.2 ± 0.2 g/L. The control group was maintained in freshwater throughout the entire experimental period.

Following the methodology described in a previous study [40], fish were sampled after 24 h of acute exposure to the designated final concentrations; they were anesthetized with MS-222 and subsequently dissected on ice. Intestinal tissues were collected from the fish for biochemical activity assays, histomorphological examination, and proteomic analysis. For each biological replicate, three fish were pooled, and each experimental group comprised three such replicates. The pool of three fish originating from the same tank was considered the experimental unit (biological replicate) for all statistical analyses. Samples designated for biochemical and proteomic analyses were immediately snap-frozen in liquid nitrogen and stored at that temperature until subsequent analysis. Samples intended for histomorphological observation were fixed in 4% buffered paraformaldehyde.

### 2.3. Histopathology

Intestinal tissues were fixed in 4% paraformaldehyde, subsequently dehydrated through a graded ethanol series, and embedded in paraffin wax. The embedded tissues were cut into 5 µm-thick sections. Following standard hematoxylin and eosin (H&E) staining, the sections were mounted with neutral balsam, and the stained tissues were visualized and photographed under an Olympus BX-51 microscope equipped with an Olympus DP25 automated imaging system (Olympus, Tokyo, Japan). Three fish per group, three sections per fish, and at least three fields per section. Because the exposure was acute (24 h), histological examination was performed as a descriptive assessment of visible morphological alterations. Quantitative morphometric measurements were not conducted.

### 2.4. Antioxidant Enzyme Activity Assay

The enzymatic activities of superoxide dismutase (SOD), catalase (CAT), and glutathione peroxidase (GSH-PX), as well as the level of malondialdehyde (MDA), a marker of lipid peroxidation, were assayed in the intestinal tissues of *O. mossambicus* using commercial detection kits (KeyGen Biotech, Nanjing, China). All measurements were carried out in strict compliance with the protocols supplied by the manufacturer. All measurements were performed spectrophotometrically: SOD activity was determined by the WST-1 method (450 nm), CAT activity by the ammonium molybdate method (405 nm), GSH-Px activity by the DTNB method (412 nm), and MDA content by the thiobarbituric acid (TBA) method (532 nm). Enzyme activities and MDA content were normalized to the total protein concentration of each sample, which was determined using a BCA protein assay kit (Thermo Fisher Scientific, Waltham, MA, USA).

### 2.5. Proteomic Analysis

#### 2.5.1. Protein Extraction and Digestion

Intestinal samples were homogenized in liquid nitrogen, then lysed in phenol-Tris-HCl buffer (pH 7.8), and subsequently centrifuged at 7100× *g* for 10 min at 4 °C. The resulting supernatant was subjected to overnight precipitation with 0.1 M ammonium acetate in methanol at −20 °C, followed by centrifugation at 12,000× *g* for 10 min at 4 °C. The resulting pellets were washed successively with methanol and acetone, briefly air-dried, and then redissolved in lysis buffer. A final centrifugation step (12,000× *g*, 10 min) was performed to obtain the clarified protein extracts. The protein concentration of each clarified extract was determined prior to digestion using a BCA protein assay kit.

#### 2.5.2. TMT Labeling and HPLC Fractionation

Protein samples (100 μL in 8 M urea containing 100 mM TEAB) were subjected to tryptic digestion at 37 °C for 4 h. Following digestion, the mixture was acidified to pH < 3, desalted using C18 cartridges, and subsequently labeled with TMT reagents for 2 h at ambient temperature. The labeling reaction was terminated by the addition of ammonia, after which the labeled samples were pooled, re-desalted, and lyophilized to dryness. The resultant fractions were separated on a C18 column (Rigol L3000 HPLC system, RIGOL Technologies, Inc., Beijing, China) employing a pH 10.0 mobile phase with a linear acetonitrile gradient ranging from 2% to 98% at 45 °C. The eluted fractions were then evaporated to dryness and reconstituted in 0.1% formic acid prior to further analysis.

#### 2.5.3. LC-MS/MS Analysis

Peptide concentrations were determined using a BCA protein assay kit (Thermo Fisher, Waltham, MA, USA) prior to loading. Peptides (1 μg) were loaded onto a C18 Nano-Trap column and analyzed on a Q Exactive™ HF-X mass spectrometer (Thermo Fisher) with a Nanospray Flex™ ion source (2.3 kV, 320 °C). MS/MS spectra were acquired via HCD fragmentation. Raw data (.raw files) were processed in Proteome Discoverer (v2.2) the UniProt reference proteome of *O. niloticus*, using the search engine implemented in Proteome Discoverer (v2.2), with trypsin as the enzyme and a maximum of two missed cleavages allowed, carbamidomethylation of cysteine as a fixed modification, and oxidation of methionine as a variable modification. Peptide-spectrum matches and protein identifications were filtered at a false discovery rate (FDR) ≤ 1% using a target-decoy strategy, and proteins were required to be identified with at least two unique peptides. Bioinformatics analysis was performed for functional annotation.

#### 2.5.4. Categorization and Bioinformatics Analysis

Gene Ontology (GO) functional annotation of the differentially expressed proteins (DEPs) was performed using BLAST2GO software (version 3.0). Proteins exhibiting a fold change (FC) greater than 1.2 or less than 0.82, with a concomitant *p*-value below 0.05, were defined as differentially expressed. The relatively permissive fold-change threshold (>1.2 or <0.82) was adopted, consistent with previous TMT-based proteomic studies, to retain the moderate but biologically relevant abundance changes that are typical of isobaric labeling workflows; *p*-values were corrected for multiple testing using the Benjamini–Hochberg procedure, and only proteins meeting both criteria were considered differentially abundant. Kyoto Encyclopedia of Genes and Genomes (KEGG) pathway enrichment analysis (available at http://www.genome.jp/kegg/ (accessed on 22 September 2026)) was employed to identify the biologically relevant pathways associated with the DEPs. A false discovery rate (FDR) of <0.05 was applied as the significance threshold to control for false-positive results.

### 2.6. Orthogonal Validation of the Proteomic Data by PRM Analysis

To corroborate the TMT-based proteomic results, the expression levels of 26 selected proteins were further quantified via parallel reaction monitoring (PRM) analysis, using the same protein samples as those employed in the original discovery experiment. Because the same biological samples were used, the PRM analysis provided an orthogonal technical validation of the TMT-based quantification rather than an independent biological validation. Protein extraction and tryptic digestion were conducted following identical procedures to those described for the TMT-based proteomic analysis. The resulting peptides were subjected to nano-electrospray ionization (NSI) and subsequently analyzed by tandem mass spectrometry (MS/MS) using a Q Exactive™ Plus mass spectrometer (Thermo Scientific, Waltham, MA, USA) coupled online to an ultra-performance liquid chromatography (UPLC) system. For MS/MS acquisition, precursor ions were fragmented using a normalized collision energy (NCE) of 27, and the resulting product ions were detected in the Orbitrap analyzer at a resolution of 17,500 (Thermo Scientific, Waltham, MA, USA). The acquired mass spectrometric data were processed using Skyline software (version 3.6).

### 2.7. Statistical Analysis

All data are presented as mean ± standard deviation (SD) based on triplicate measurements (*n* = 3 per group), with the pool of three fish from the same tank serving as one biological replicate (experimental unit). The normality of the data distribution was verified using the Shapiro–Wilk test prior to analysis. Statistical comparisons among the four experimental groups, which constitute a 2 × 2 factorial design (salinity × alkalinity), were performed using one-way analysis of variance (ANOVA) followed by Tukey’s post hoc test for pairwise comparisons, with a significance level of *p* < 0.05; in addition, the main effects of salinity and alkalinity and their interaction were evaluated by two-way ANOVA. Given the relatively small sample size (*n* = 3) of the biochemical assays, the corresponding results should be interpreted with appropriate caution. All analyses were conducted using SPSS software (version 22.0).

## 3. Results

### 3.1. Intestine Histological Observation

**Figure 1:**
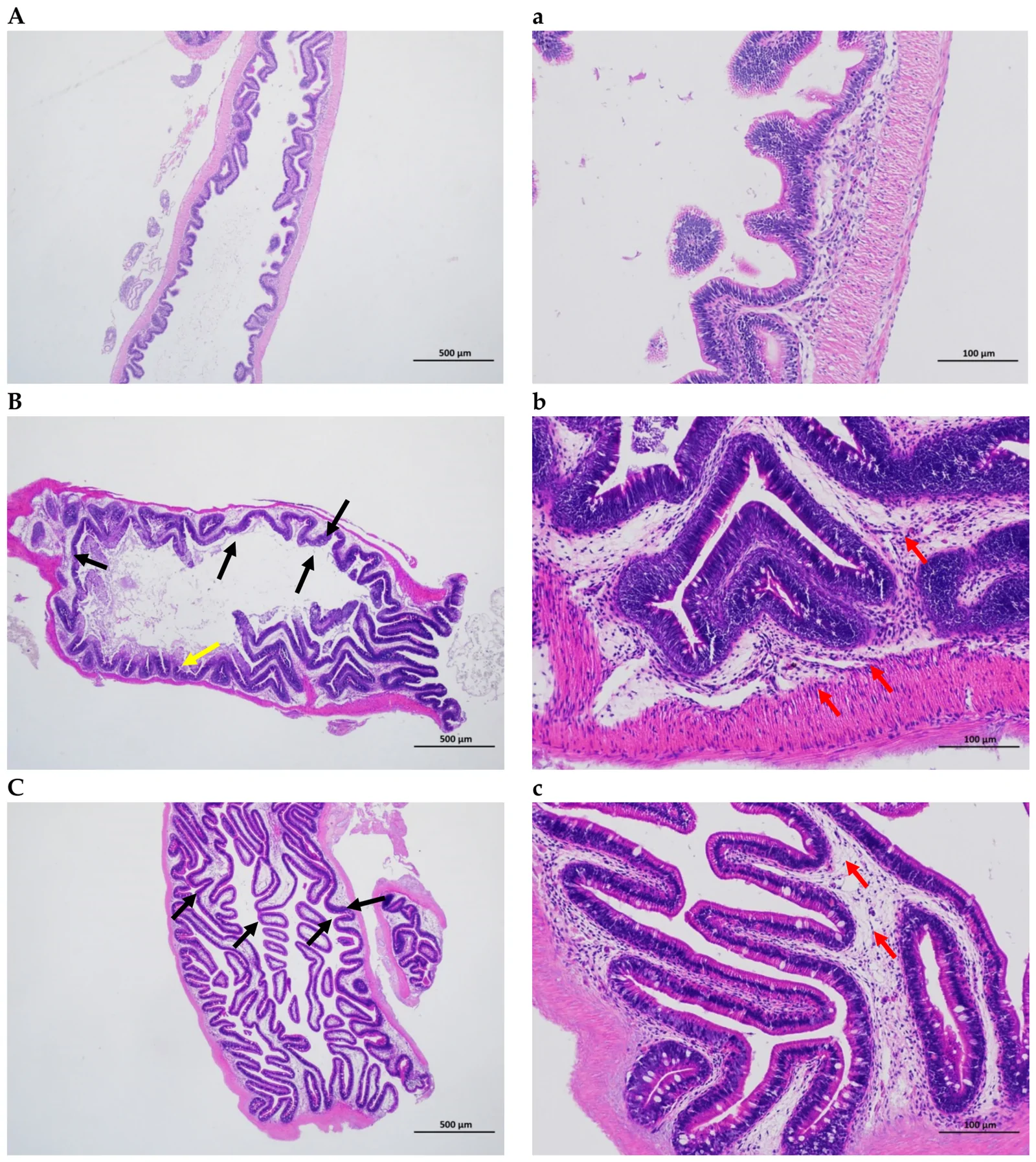

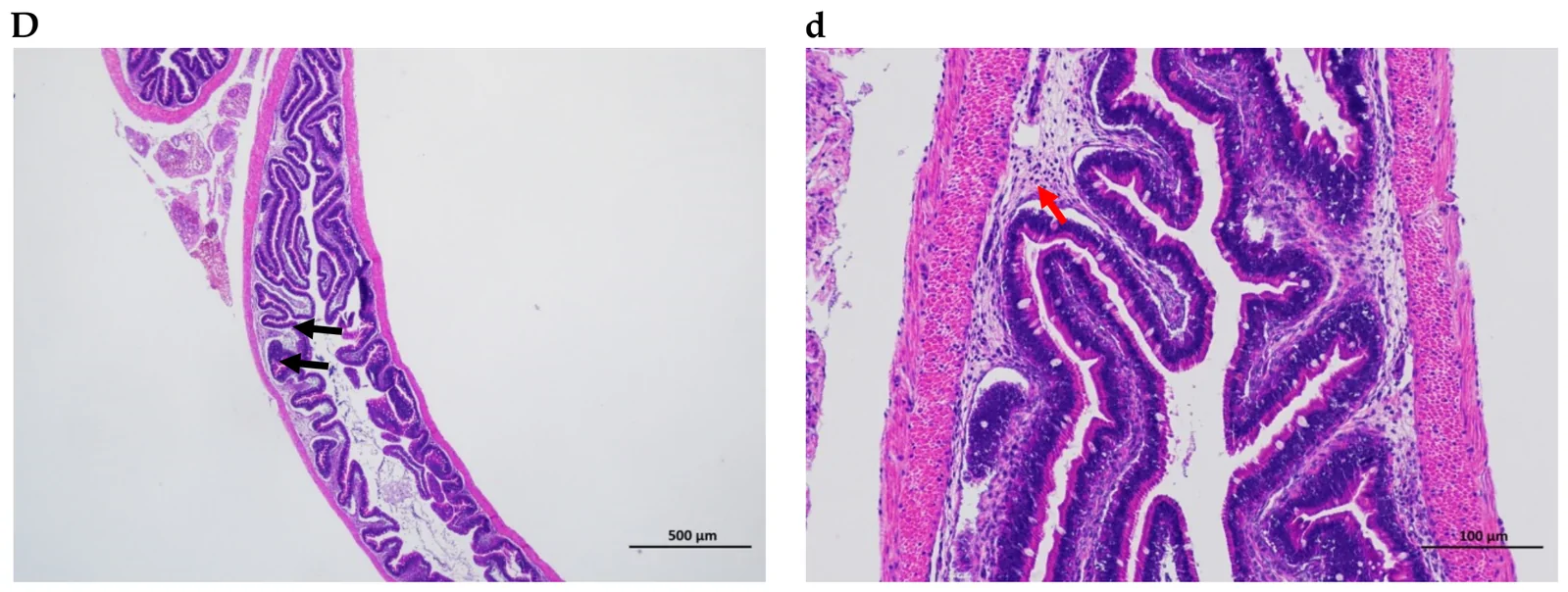
The histological observation of *O. mossambicus* intestine tissue under salinity, alkalinity and saline–alkalinity. (HE stain, **A**–**D**: ×40, **a**–**d**: ×200) (**A**,**a**), the intestine of the control group; (**B**,**b**), the intestine of the salinity stress group; (**C**,**c**), the intestine of the alkalinity stress group; (**D**,**d**), the intestine of the saline–alkalinity stress group. Note: black arrow: loose lamina structure and light staining; red arrow: a few inflammatory cells scattered in the infiltration; yellow arrow: autolysis at the top of the mucous villi.

Histological observations suggested visible morphological alterations in the intestinal tissue after acute stress. The intestines stained with hematoxylin and eosin from the control group and stressed group were shown in Figure 1. In the control group, the intestinal mucosa epithelium was intact, and the epithelial cells were closely arranged and normal in shape. After 24 h of salinity stress, the intestinal tissue was extensively and lightly edematous, with a loose lamina structure and light staining, accompanied by a few inflammatory cells scattered in infiltration, and autolysis at the top of the mucous villi. In the alkalinity and saline–alkali stress groups, the intestinal tissue was extensively edematous, and the lamina was loose, with light staining, accompanied by a few inflammatory cells with scattered infiltration.

### 3.2. Antioxidant Enzyme Activity

**Figure 2:**
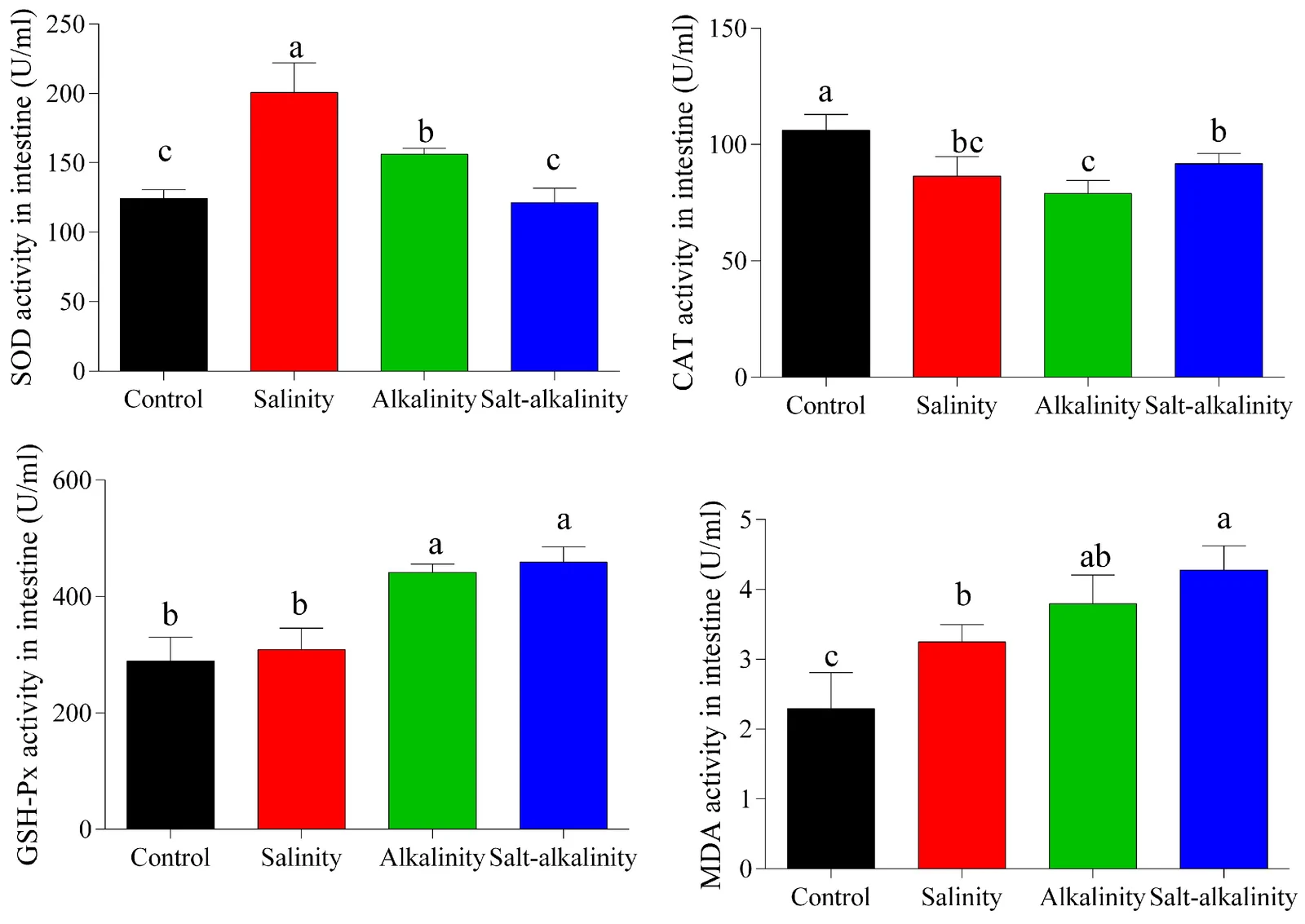
Activities of SOD, CAT, GSH-Px, and MDA content in the intestine of *O. mossambicus* under three osmotic stresses (*n* = 3). Different lowercase letters indicate significant differences among the groups (*p* < 0.05).

Intestinal oxidative stress responses were evaluated by measuring the activities of SOD, CAT and GSH-Px, as well as MDA levels, in the gut tissues of *O. mossambicus* subjected to salinity, alkalinity and combined saline–alkalinity stress (Figure 2). Relative to the control group, SOD activity was significantly elevated under both salinity (*p* < 0.05) and alkalinity (*p* < 0.05) stress, whereas no significant change was observed in the saline–alkalinity group (*p* > 0.05). In contrast, CAT activity exhibited a marked reduction under all three stress conditions compared with the control (*p* < 0.05), with the lowest activity recorded in the alkalinity group (*p* < 0.05). GSH-Px activity showed a significant increase in the alkalinity and saline–alkalinity groups (*p* < 0.05), but remained unchanged under salinity stress (*p* > 0.05). Assessment of MDA levels, as an indicator of lipid peroxidation, revealed significant increases in all three stressed groups relative to the control (*p* < 0.05), and the highest MDA content was recorded in the saline–alkalinity group (*p* < 0.05). Pairwise comparisons among the treatment groups (Duncan’s multiple range test) further revealed that SOD activity was significantly higher in the salinity group than in the alkalinity (*p* < 0.01) and saline–alkalinity (*p* < 0.05) groups, and that SOD activity in the alkalinity group was in turn significantly higher than in the saline–alkalinity group (*p* < 0.01); that GSH-Px activity was significantly higher in both the alkalinity and saline–alkalinity groups than in the salinity group (*p* < 0.05), whereas no significant difference was detected between the alkalinity and saline–alkalinity groups (*p* > 0.05); that CAT activity was significantly lower in the saline–alkalinity group than in the alkalinity group (*p* < 0.05); and that MDA content in the saline–alkalinity group was significantly higher than that in the salinity group (*p* < 0.05), whereas the salinity and alkalinity groups did not differ significantly from each other (*p* > 0.05). Taken together, these results indicate that salinity stress elicited the most pronounced induction of intestinal SOD, whereas combined saline–alkalinity stress caused the most severe lipid peroxidation accompanied by a blunted SOD response; CAT activity was suppressed under all three stress conditions, most markedly under alkalinity stress. These between-treatment differences are indicated by lowercase letters in Figure 2.

### 3.3. Proteomic Profiles in the Intestine of Osmotically Stressed

**Figure 3:**
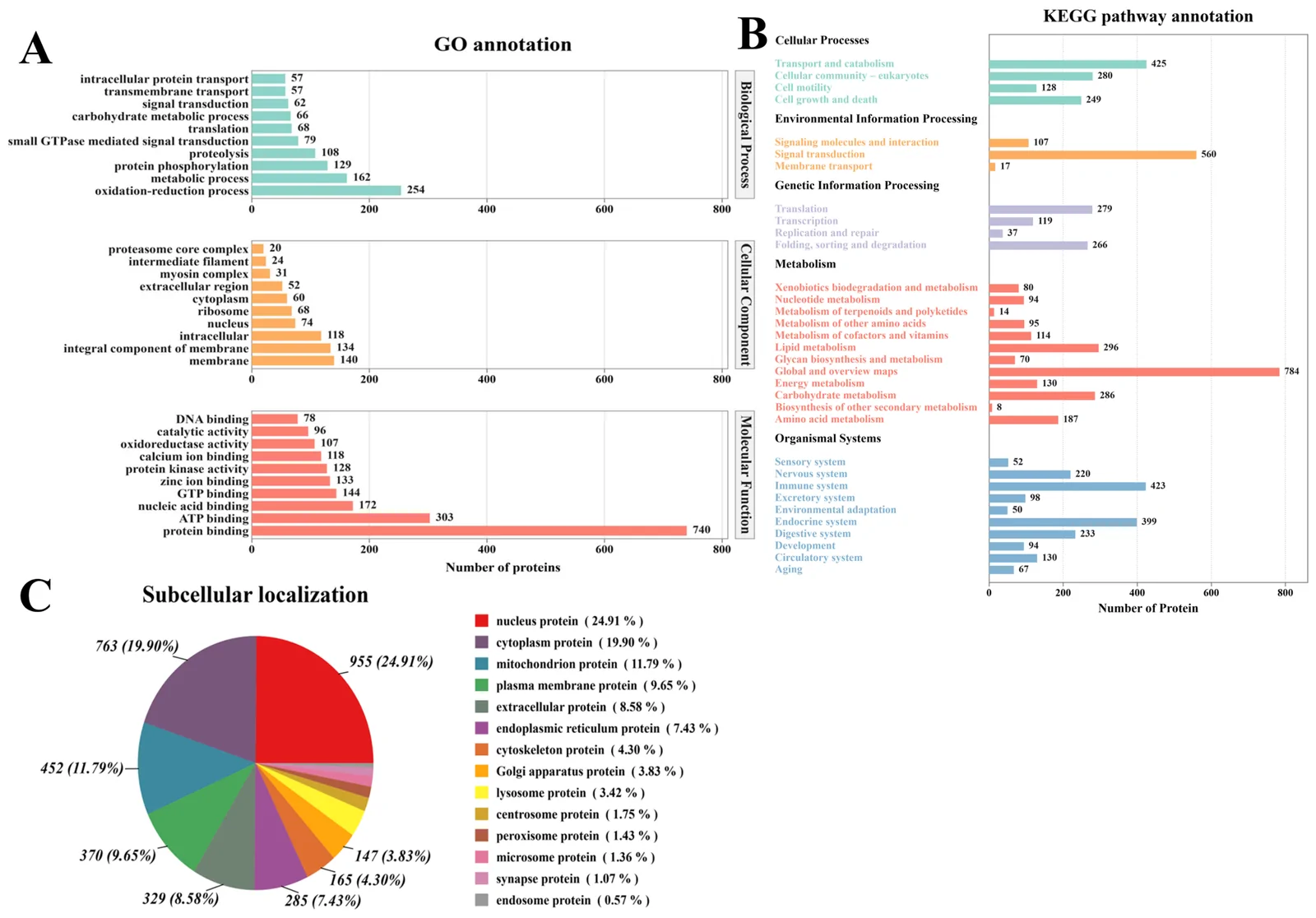
GO annotation, KEGG pathway enrichment and subcellular localization analysis of quantitative proteins. (**A**): GO annotation of quantitative proteins; (**B**): KEGG pathway enrichment of quantitative proteins; (**C**): The subcellular localization of identified proteins. The different colors indicate the localization of the identified proteins.

To evaluate the effects of osmotic stress on the intestinal tissues of *O. mossambicus*, TMT-based proteomic analysis was performed on samples exposed to salinity, alkalinity, and combined saline–alkalinity stress. In total, 371,633 acquired MS/MS spectra were matched to 65,272 spectra, leading to the identification of 5225 proteins from 28,743 unique peptides. Among the 5225 identified proteins, 5197 were reliably quantified and thus retained for further analysis. Gene Ontology (GO) annotation, Kyoto Encyclopedia of Genes and Genomes (KEGG) pathway enrichment, and subcellular localization prediction were subsequently performed to functionally characterize the 5197 quantified proteins (Figure 3). Based on the biological process (BP) ontology, these quantified proteins were implicated in diverse biological functions, including oxidation-reduction, metabolic processes, protein phosphorylation, and other relevant pathways. KEGG pathway enrichment analysis revealed that the quantified proteins were associated with five major categories: cellular processes, environmental information processing, genetic information processing, metabolism, and organismal systems. Subcellular localization analysis further indicated that nuclear, cytoplasmic, and mitochondrial proteins accounted for 24.91%, 19.9%, and 11.79% of the quantified proteins, respectively.

#### 3.3.1. Proteomic Profiles in Intestine of Salinity-Stressed

**Figure 4:**
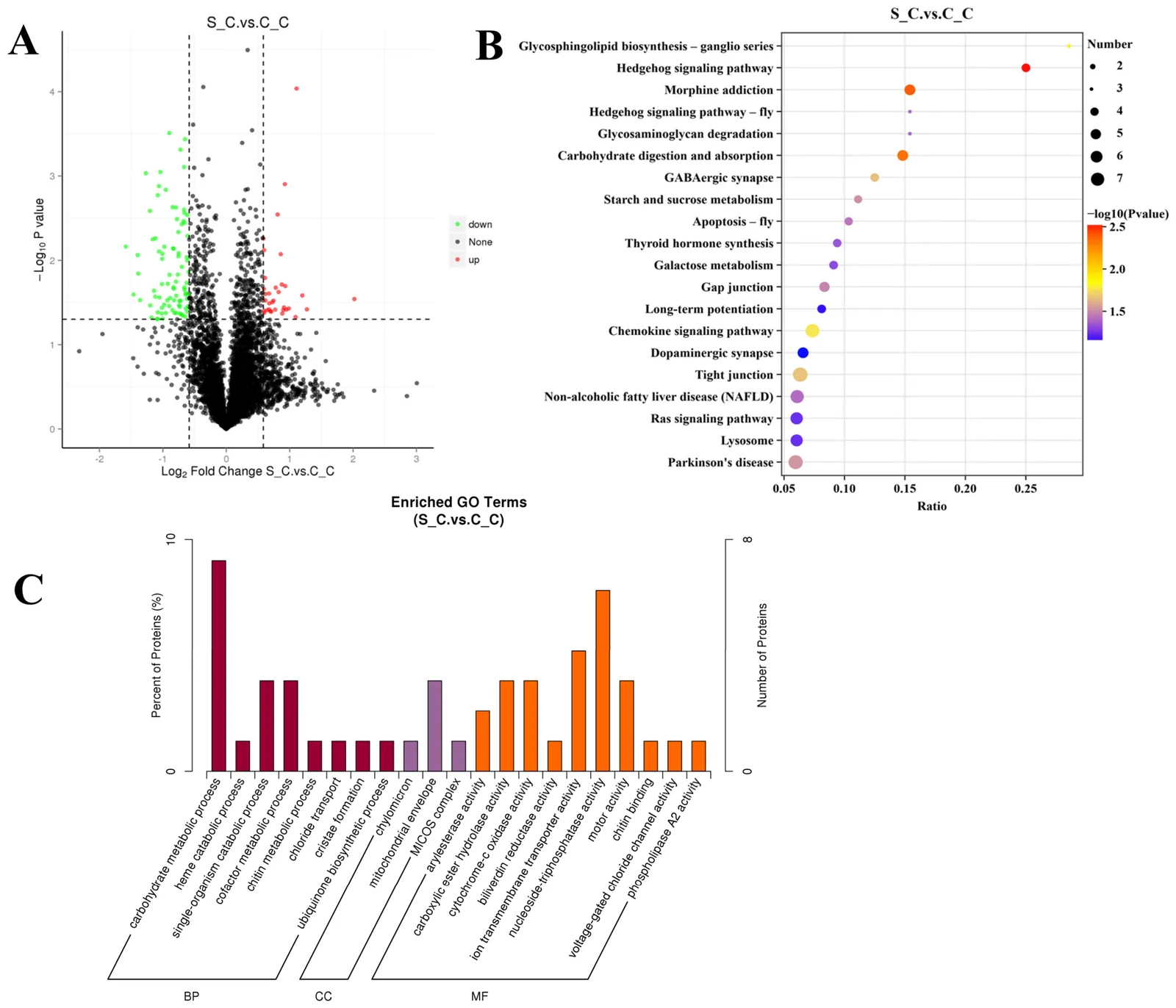
Functional analysis of differentially expressed proteins in the S_C vs. C_C. (**A**): Volcano plots of DEPs after salinity stress; (**B**): KEGG pathway enrichment analysis of DEPs; (**C**): GO-based enrichment analysis of DEPs.

To gain further insight into the alterations and underlying mechanisms of salinity stress responses in the intestine of *O. mossambicus*, global proteomic profiling was performed on salinity-exposed intestinal tissues using the TMT-based quantitative approach. In total, more than 5000 proteins were successfully identified and quantified. Among these, 120 proteins exhibited significant alterations in abundance in response to salinity treatment, based on the criteria of *p* < 0.05 and a fold change (FC) > 1.2 or <0.82. Of these differentially expressed proteins (DEPs), 34 were up-regulated and 86 were down-regulated (Figure 4). Functional annotation revealed that several DEPs were associated with osmoregulatory processes, including solute carrier family 25 member 24 (SLC25A24), solute carrier family 25 member 12 (SLC25A12), and chloride voltage-gated channel 7 (CLCN7), further corroborating the impact of salinity stress on intestinal ion transport. Gene Ontology (GO) terms for biological processes and KEGG pathway enrichment analyses were subsequently retrieved for the identified DEPs. Based on the biological process (BP) classification, the DEPs were implicated in a broad spectrum of functional categories, including metabolic processes, catabolic processes, chloride transport, and the ubiquinone biosynthetic pathway. KEGG pathway analysis further identified 15 significantly enriched pathways (*p* < 0.05), with marked enrichment observed in tight junctions (map 04530) and Parkinson disease (map 05012). It should be noted that disease-related KEGG terms such as Parkinson disease do not imply the occurrence of disease in fish; they reflect the enrichment of evolutionarily conserved molecular components (e.g., mitochondrial, proteasomal, and synaptic proteins) that are shared between disease-related pathways and the cellular processes perturbed by osmotic stress.

#### 3.3.2. Proteomic Profiles in Intestine of Alkalinity-Stressed

**Figure 5:**
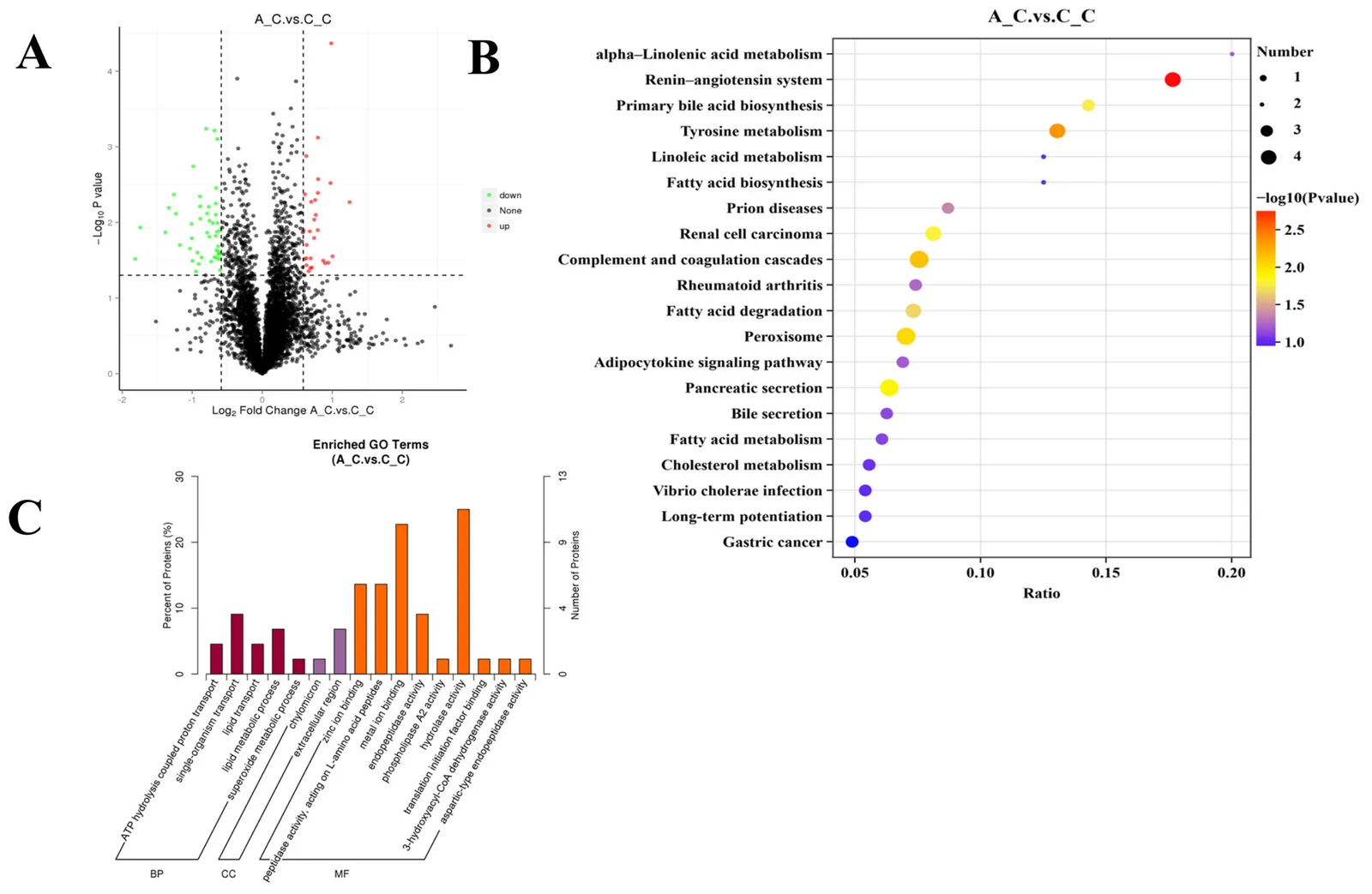
Functional analysis of differentially expressed proteins A_C vs. C_C. (**A**): Volcano plots of DEPs after salinity stress; (**B**): KEGG pathway enrichment analysis of DEPs; (**C**): GO-based enrichment analysis of DEPs.

In total, 5197 proteins were present in all samples and thus could be quantified. Of these proteins, 73 were identified in the alkalinity-stressed group, with 45 being downregulated and 28 being upregulated according to a *p*-value < 0.05 and FC > 1.2 or FC < 0.82. The identified proteins were initially classified into two clusters based on their expression patterns, and subsequently subjected to Gene Ontology (GO) annotation for functional characterization. In total, 73 differentially expressed proteins (DEPs) were found to be associated with 24 biological process terms, 32 molecular function terms, and 4 cellular component terms. The top ten significantly represented biological processes encompassed three major functional categories: transport-related processes (including ATP hydrolysis-coupled proton transport, single-organism transport, lipid transport, and transmembrane transport); metabolic processes (comprising lipid metabolic process, superoxide metabolic process, lipid catabolic process, fatty acid metabolic process, and lipoprotein metabolic process); and translation-related processes (translational initiation). KEGG pathway enrichment analysis was additionally conducted to elucidate the signaling pathways affected in response to alkalinity stress. This analysis revealed that the 73 DEPs were mapped to a total of 94 KEGG pathways. Nine pathways that changed significantly after exposure to alkalinity stress (Figure 5), including the renin-angiotensin system (map 04614), tyrosine metabolism (map 00350), complement and coagulation cascades (map 04610), peroxisomes (map 04146), pancreatic secretion (map 04972), renal cell carcinoma (map 05211), primary bile acid biosynthesis (map 00120), fatty acid degradation (map 00071), and prion diseases (map05020). As noted above, the disease-related terms (renal cell carcinoma, prion diseases) represent the enrichment of conserved molecular components rather than evidence of pathological processes in the fish.

#### 3.3.3. Proteomic Profiles in Intestine of Saline–Alkalinity Stressed

**Figure 6:**
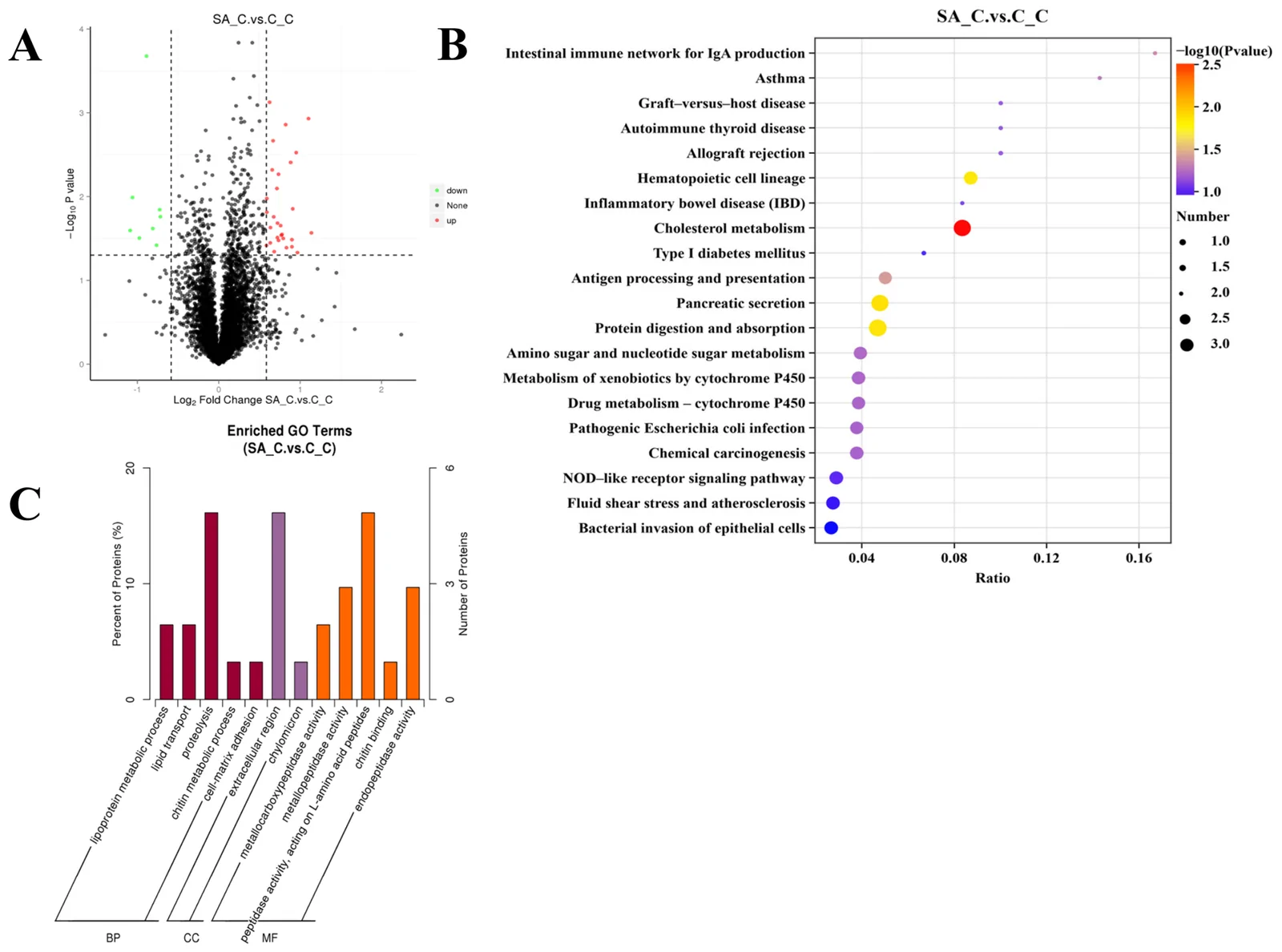
Functional analysis of differentially expressed proteins SA_C vs. C_C. (**A**): Volcano plots of DEPs after salinity stress; (**B**): KEGG pathway enrichment analysis of DEPs; (**C**): GO-based enrichment analysis of DEPs.

Among the 5197 identified and quantified proteins, 37 exhibited significantly altered abundance in the intestine following combined saline–alkalinity treatment, of which 29 were up-regulated and 8 were down-regulated. The functional classification of these 37 differentially expressed proteins (DEPs), as determined by Gene Ontology (GO) annotation and KEGG pathway enrichment analysis, is presented in Figure 6. GO biological process annotation revealed that the up-regulated proteins were predominantly associated with proteolysis and antigen processing and presentation, whereas the down-regulated proteins were mainly enriched in lipoprotein metabolic processes. KEGG pathway analysis further demonstrated that the 37 DEPs were mapped to 86 pathways, among which six were significantly enriched following saline–alkalinity stress, namely cholesterol metabolism (map04979), pancreatic secretion (map04972), protein digestion and absorption (map04974), hematopoietic cell lineage (map04640), antigen processing and presentation (map04612), and the intestinal immune network for IgA production (map04672). Moreover, KEGG analysis indicated that the up-regulated proteins were predominantly involved in immune and inflammatory responses—including antigen processing and presentation, intestinal immune network for IgA production, pathogenic Escherichia coli infection, and asthma—whereas the down-regulated proteins were primarily associated with metabolic pathways, such as cholesterol metabolism, metabolism of xenobiotics by cytochrome P450, drug metabolism by cytochrome P450, and ascorbate and aldarate metabolism.

### 3.4. Comparative Proteomics Between the Different Osmotic Stresses

**Figure 7:**
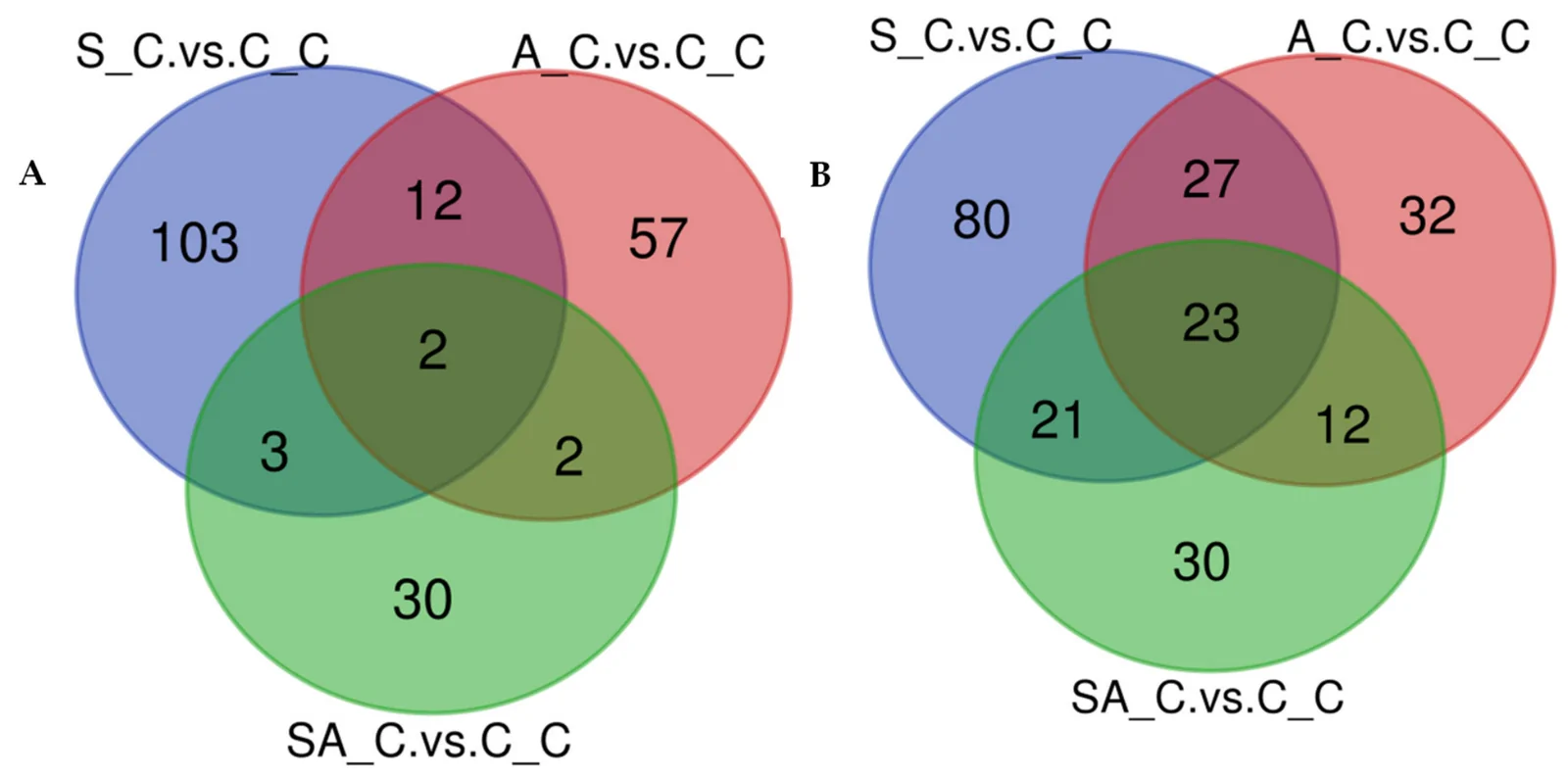
Number of DEPs and Venn diagram of the overlap of the different groups. (**A**): DEPs number and Venn diagram of overlap of the S_C vs. C_C, A_C vs. C_C and SA_C vs. C_C groups; (**B**): KEGG pathways number and Venn diagram of overlap of the S_C vs. C_C, A_C vs. C_C and SA_C vs. C_C groups.

Comparative analysis of the proteomes of salinity, alkalinity, and saline–alkalinity from control conditions revealed two proteins exclusively found in the three treatment groups, and 12 proteins were found in the salinity and alkalinity stress groups but not in the saline–alkalinity group. Furthermore, 103, 57, and 30 differentially expressed proteins (DEPs) were identified exclusively in the salinity, alkalinity, and combined saline–alkalinity stress groups, respectively (Figure 7A). Meanwhile, 23 KEGG pathways were commonly enriched across all three treatment groups, whereas 80, 32, and 30 pathways were uniquely enriched in the salinity, alkalinity, and saline–alkalinity groups, respectively (Figure 7B). To further characterize the effects of distinct osmotic stressors on the intestinal proteome of *O. mossambicus*, comparative analyses were performed among the salinity, alkalinity, and combined saline–alkalinity treatment groups. Relative to the salinity-stressed group, the alkalinity-stressed and saline–alkalinity-stressed groups exhibited 57 and 120 DEPs, respectively. In addition, when compared with the alkalinity-stressed group, the saline–alkalinity-stressed group yielded 27 DEPs, of which 19 were up-regulated and 8 were down-regulated. Between the alkalinity- and salinity-stressed groups, 12 pathways were significantly enriched, of which 5 were associated with disease-related pathways and 1 was involved in oxidative phosphorylation. Between the saline–alkalinity- and salinity-stressed groups, nine pathways were significantly enriched, including those related to Parkinson disease, oxidative phosphorylation, cholesterol metabolism, and Alzheimer disease, among others. These disease-related pathway names likewise refer to conserved eukaryotic protein sets and should not be interpreted as pathological processes occurring in the fish. Furthermore, relative to the alkalinity-stressed group, 11 pathways were significantly enriched in the saline–alkalinity-stressed group, including autoimmune thyroid disease, the intestinal immune network for IgA production, and neuroactive ligand-receptor interaction, among others.

### 3.5. Orthogonal Validation of the Proteomic Data by PRM

**Figure 8:**
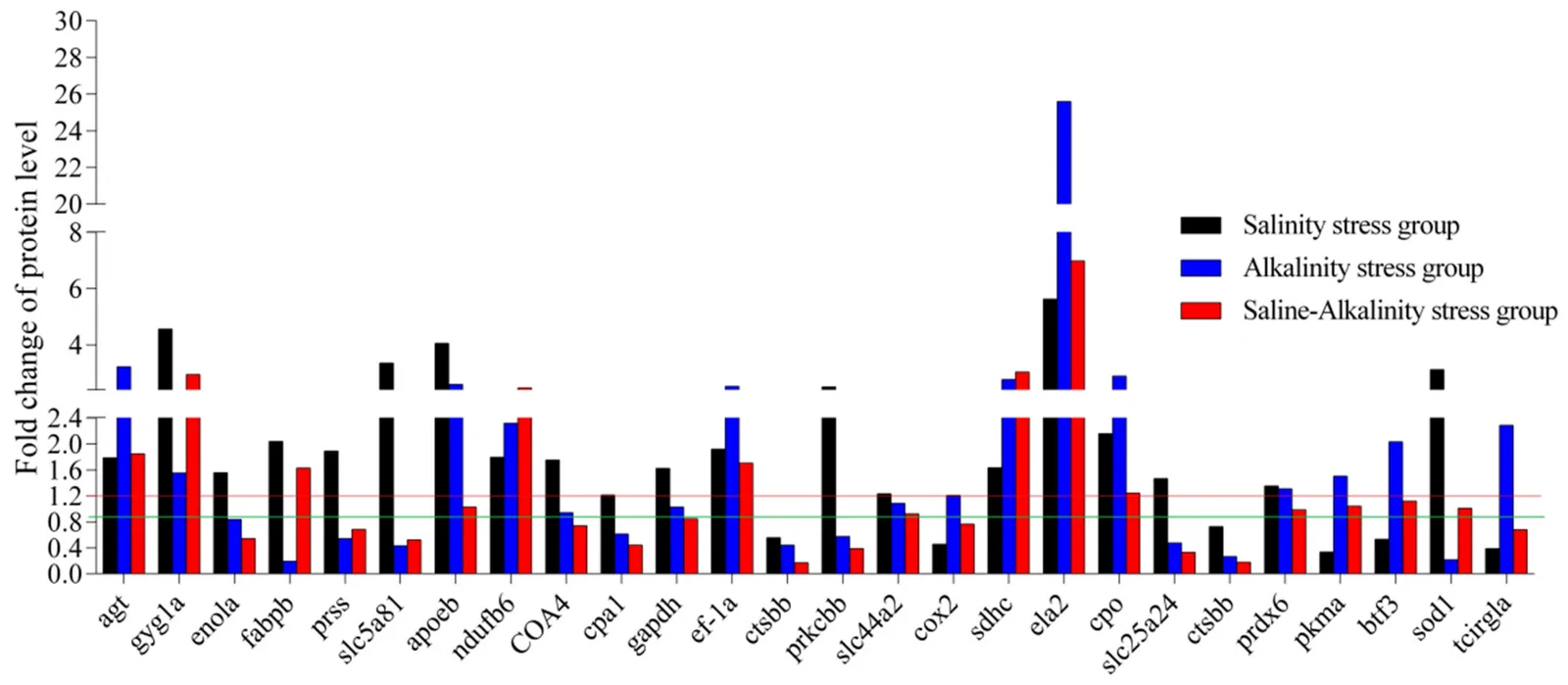
Relative quantification of differentially expressed proteins in the intestine of *O. mossambicus* under salinity, alkalinity and saline–alkalinity stress using PRM analysis. (*n* = 3 biological replicates per group) The green line represents a fold change of 0.82, and the red line represents a fold change of 1.2. A fold change >1.2 or <0.82 indicates that the protein was significantly differentially expressed.

**Figure 9:**
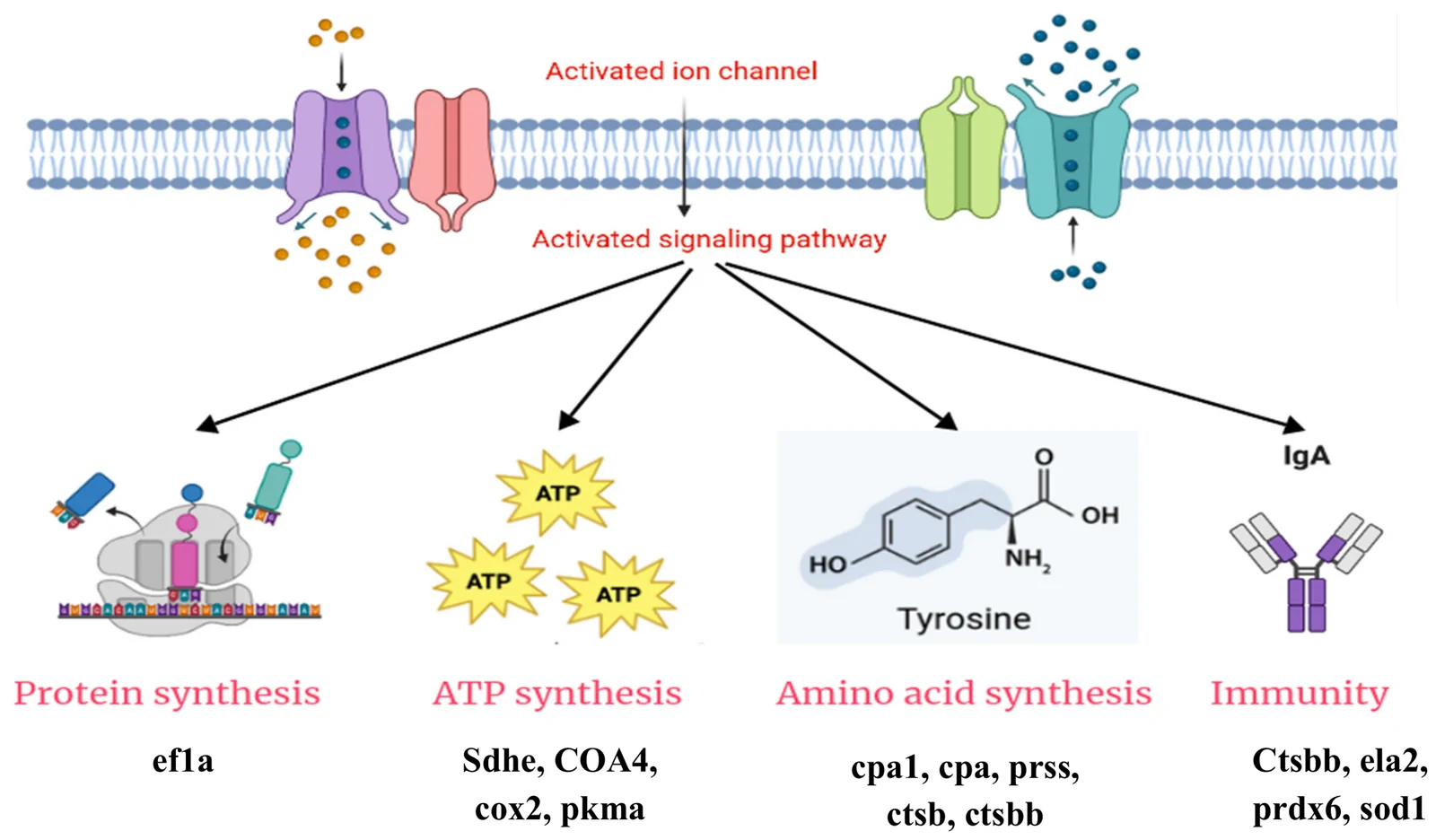
Proposed signaling pathways in the intestine of *O. mossambicus* under salinity, alkalinity, and combined saline–alkalinity stress. Enriched pathways are labeled in red. Pathway names are presented as summary labels, and representative identified proteins are listed for each pathway. The names, functional roles, and positions of the identified proteins within these biological processes are indicated. A complete list and detailed annotations are provided in Supplementary Table S1.

A total of 26 proteins were validated across the three osmotic stress groups, and the parallel reaction monitoring (PRM) results confirmed that the expression trends of all selected proteins were consistent with the TMT-based proteomic data (Figure 8). Among these 26 proteins, six—*agt*, *gyg1a*, *ndufb6*, *ef-1a*, *sdhc*, and *ela2*—were up-regulated in response to all three osmotic stress conditions. In contrast, *enola*, *prss*, *slc5a81*, *cpa1*, *prkcbb*, and *slc25a24* were down-regulated in the alkalinity and combined saline–alkalinity stress groups. Additionally, *btf3*, *tcirgla*, and *pkma* exhibited up-regulation specifically under alkalinity stress, whereas *ctsbb* was consistently down-regulated across all three stress conditions. Overall, the protein expression changes quantified by PRM were largely in agreement with the findings obtained from the TMT-based proteomic analysis. Because the PRM analysis used the same biological samples as the discovery experiment, this consistency reflects orthogonal analytical validation of the TMT-based quantification. The proposed signaling pathways implicated in the intestinal responses to the three osmotic stresses are summarized in Figure 9.

## 4. Discussion

### 4.1. Histological Response Under Three Osmotic Stresses

The intestinal tract serves multiple physiological roles in fish, including nutrient absorption and digestion, which profoundly influence overall metabolism and systemic physiological homeostasis [41]. Among all internal organs, the intestine possesses the largest surface area in direct contact with the external environment. Despite being anatomically internal, the intestinal lumen is in close proximity to the ambient water and thus plays a critical role in maintaining water and mineral balance in teleosts. Through sensing the permeability of ingested seawater and regulating drinking rates as well as water and ion absorption, the intestinal system of teleosts constitutes an essential component of their adaptive response to saline environments. To achieve effective osmoregulation during transitions between freshwater and marine environments, euryhaline fish are capable of extensively remodeling their intestinal architecture [42]. In the present study, exposure to salinity, alkalinity, and combined saline–alkalinity stress was found to induce notable histopathological alterations in the intestinal tissues of *O. mossambicus*, including edema, autolysis, and necrosis of the lamina propria within the intestinal villi. Collectively, these observations underscore the critical role of the intestine in mediating responses to osmotic stress.

### 4.2. Response to Cope with Osmotic Stress Fluctuation

Osmotic perturbation represents a critical environmental constraint influencing the growth and survival dynamics of teleost species [12,43]. Shifts in ambient salinity disrupt the physiological equilibrium of aquatic organisms, triggering the excessive generation of reactive oxygen species (ROS) [44]; this cascade subsequently promotes the oxidation of lipids and proteins, induces oxidative injury to cellular membranes and nuclear genetic material, and impairs the functional activity of various enzymatic systems [45]. Consistent with these findings, prior investigations have established that stressful fluctuations in environmental parameters compromise normal cell function, ultimately precipitating lipid peroxidation [46]. Indeed, oxidative challenge is a primary pleiotropic consequence of hyperosmotic conditions or hypoxic exposure in aquatic taxa [47]. This state, in turn, stimulates the activation of key antioxidative enzymes—including SOD, CAT, and GPX—as a compensatory mechanism to counter the elevated ROS burden, which is inherently linked to DNA fragmentation and the progressive loss of cellular integrity [48]. Superoxide dismutase catalyzes the dismutation of superoxide radicals into oxygen and hydrogen peroxide, thereby functioning as an essential frontline antioxidant that affords protection to cells under aerobic conditions. Concurrent fluctuations in both thermal and osmotic regimes within the aquatic environment are known to provoke substantial production of oxygen-derived free radicals in fish. As an immediate component of the antioxidant defense network, SOD facilitates the conversion of these free radicals into hydrogen peroxide, which is subsequently reduced to water and molecular oxygen by catalase; this coordinated action effectively mitigates peroxide-mediated cytotoxicity and safeguards the homeostasis of the intracellular milieu [28]. Moreover, malondialdehyde (MDA) content, a widely recognized proxy for the extent of lipid peroxidation [49], is significantly elevated under such adverse environmental circumstances [50].

Our results demonstrated that in the intestine of *O. mossambicus*, exposure to salinity or alkalinity individually elevated the activities of SOD and GSH-Px and increased MDA levels while concurrently reducing CAT activity. Under combined saline–alkaline stress, however, GSH-Px and MDA were similarly increased, whereas both SOD and CAT were diminished. These observations are consistent with the report of Xavier et al. [51], who found that short-term high-salinity challenge enhances antioxidative capacity in Nile tilapia. Oxidative stress arises when the overproduction of reactive oxygen species (ROS) exceeds the buffering capacity of the endogenous antioxidant system, thereby disturbing cellular homeostasis and potentially leading to cell death [52,53,54]. If the oxidative insult is excessively severe or prolonged, the resulting accumulation of oxygen free radicals overwhelms the antioxidative enzymes, triggering apoptotic cascades and a consequent sharp decline in enzyme activities [55]. Supporting this view, Xu et al. [56] documented that chronic high-salinity exposure impairs the antioxidative defense in Nile tilapia, emphasizing that stress duration critically determines the net antioxidant response. The alterations observed in antioxidant-related responses in the present study are consistent with previous findings in tilapia, where chronic exposure to 25 ppt salinity affected antioxidant capacity and physiological stability [25]. Although the exposure duration differed substantially between the two studies (24 h here versus 60 days in Firdaus et al. [25]), both indicate that salinity can induce oxidative and physiological stress responses in tilapia. In line with this, recent findings in red tilapia demonstrated that acute salinity exposure induces dynamic changes in antioxidant defenses, including SOD, CAT, and GSH-Px, accompanied by increased MDA levels and disruption of ion homeostasis [57].

### 4.3. Intestine Osmoregulation Under Osmotic Stress

Aquatic vertebrates are constantly challenged by osmotic gradients between their internal body fluids and the external medium. In hypo-osmotic environments, fish face a continuous net influx of water that must be eliminated, whereas those occupying hyperosmotic marine habitats undergo passive dehydration [58]. To circumvent these challenges, euryhaline teleosts have evolved a suite of physiological strategies, including the ingestion of ambient water (in hyperosmotic conditions) and the active absorption of monovalent ions along the esophagus. This process lowers the osmolality of the ingested fluid, thereby promoting water absorption through aquaporin-mediated pathways in the intestinal epithelium [59,60,61]. The intestine is one of the tissues most intimately exposed to the external environment and also serves as a major organ involved in both immune defense and osmotic regulation [62]. Nevertheless, intestinal contributions to osmoregulation are inherently complex and depend on both species-specific factors and the particular type of osmotic challenge under investigation [63,64,65]. To date, most studies on teleost osmoregulation have concentrated on the gill [11,66] and the kidney [67], while the role of the intestine has, with few exceptions, received comparatively little attention. Advances in bioinformatics have propelled the extensive adoption of omics approaches across diverse biological disciplines. Transcriptomic analyses of intestinal responses to varying salinity acclimation have been conducted in several teleost species [42,68,69,70], significantly deepening our understanding of the osmoregulatory events occurring in the gut. However, it is now well recognized that mRNA abundance accounts for only 40–60% of the observed variation in protein levels [71,72]; consequently, direct protein measurement generally affords a more accurate reflection of cellular functional states than transcript-based inferences. The application of omics technologies, particularly proteomics, has facilitated the discovery of biochemical pathways that underpin phenotypic differences [73]. As one of the most powerful analytical tools, proteomics offers the distinct advantage of capturing more direct molecular responses relative to gene-level analyses [74]. Indeed, proteomic profiling has already been employed to investigate protein-level adaptations to hypersaline exposure in multiple fish species [37,38,75]. In the present study, we performed and analysed intestinal proteomic profiles of *O. mossambicus* subjected to salinity, alkalinity, and combined saline–alkaline stress, aiming to unravel the molecular osmoregulatory mechanisms operative in the intestinal tract under diverse osmotic pressures. Relative to the control group, we identified 120, 73, and 37 significantly differentially expressed proteins (DEPs) in the intestine following salinity, alkalinity, and saline–alkaline challenges, respectively. Among these DEPs, several were annotated as being involved in ion transport and osmoregulation, including sodium-coupled monocarboxylate transporter 1 (slc5a8l), solute carrier family 44 member 2 (slc44a2), V-type proton ATPase 116 kDa subunit a (tcirg1a), ATPase H^+^ transporting accessory protein 2 (atp6ap2), and ATPase H^+^ transporting accessory protein 1b (atp6ap1b). Exposure to osmotic stress is known to elevate reactive oxygen species (ROS) production, which in turn provokes oxidative stress and ultimately triggers apoptotic pathways, as documented in previous work [76]. To counteract ROS-mediated cellular damage, organisms have developed a sophisticated scavenging system comprising both non-enzymatic antioxidants and antioxidative enzymes [77]. Consequently, the activities of these enzymes are routinely employed as reliable biomarkers for assessing oxidative stress elicited by osmotic challenges. Among them, superoxide dismutase 1 (SOD1) was observed to be upregulated in the intestine of *O. mossambicus*; this metalloenzyme plays an indispensable role in catalyzing the dismutation of superoxide radicals into molecular oxygen and hydrogen peroxide, thereby safeguarding cells against oxidative injury [78]. In addition, oxygenases are critically involved in maintaining cellular homeostasis by alleviating oxidative stress induced by various exogenous stimuli [79], including heat shock [80] and hypoxia [81]. In the present study, SOD1 protein levels exhibited contrasting regulation patterns under different stress conditions: an increase was noted following salinity exposure, whereas a decrease was observed under alkalinity stress. Notably, in the combined saline–alkalinity group, the SOD1 level did not differ significantly from that of the control group. KEGG enrichment analysis of the identified DEPs yielded 151, 94, and 86 enriched pathways in the salinity, alkalinity, and combined saline–alkalinity stress groups, respectively. Of these, 15, 9, and 6 pathways reached statistical significance in the corresponding groups. Under salinity stress, the Hedgehog (Hh) signaling pathway emerged as the most significantly enriched, exhibiting the highest level of significance. The Hh signaling pathway is a critical regulator of embryonic patterning, physiological programming, tissue regeneration, and cell proliferation in vertebrates [82]. Aberrant Hh signaling has been implicated in various pathological conditions, ranging from tumorigenesis to metabolic disorders [83,84,85,86,87,88]. In addition, several other significantly enriched pathways in the salinity-stressed intestine of *O. mossambicus* were associated with energy metabolism (e.g., carbohydrate digestion and absorption, starch and sucrose metabolism, and glycosaminoglycan degradation), disease and immunity (e.g., morphine addiction, chemokine signaling, Parkinson’s disease, and non-alcoholic fatty liver disease), hormone synthesis and secretion (thyroid hormone synthesis), and apoptosis. Salinity fluctuations are known to elevate oxygen consumption and energy demands, accelerate metabolic rates, disrupt normal physiological functions, and compromise immune defense in fish [89,90]. Moreover, Jiang et al. [11] demonstrated that carbohydrate metabolism serves as the primary energy source for ion and osmotic regulation. Maintaining a homeostatic balance between epithelial cell proliferation and apoptosis is fundamental for preserving epithelial functions, such as permeability regulation, inflammatory responses, and nutrient absorption [91]. Apoptosis is central to epithelial organization and cell turnover; accordingly, disruptions in enterocyte apoptotic pathways have been linked to villus atrophy, epithelial hyperplasia, and impaired absorptive capacity [92].

Under alkalinity stress, a set of pathways—including the renin-angiotensin system, tyrosine metabolism, complement and coagulation cascades, peroxisomes, pancreatic secretion, renal cell carcinoma, primary bile acid biosynthesis, fatty acid degradation, and prion disease—were significantly enriched in the intestinal proteome of *O. mossambicus*. It should be emphasized that the disease-related terms in this list (renal cell carcinoma, prion disease) denote conserved molecular components enriched among the DEPs and do not indicate pathological conditions in the experimental fish. When euryhaline fish transition from freshwater to hyperosmotic environments, systemic homeostasis is sustained through multiple hormonally regulated responses [93]: activation of the renin-angiotensin system triggered by transient hypovolemia resulting from branchial water loss; angiotensin II-mediated stimulation of drinking, which facilitates intestinal water and electrolyte uptake; cortisol-dependent upregulation of gill Na^+^-K^+^-ATPase activity to eliminate excess Na^+^ and Cl^−^; and renal adjustments characterized by reduced glomerular filtration rate (GFR) and diminished urine production. The enrichment of tyrosine metabolism under alkaline conditions is particularly noteworthy, given the recognized contribution of free amino acids to osmotic regulation [94,95]. This observation suggests that *O. mossambicus* modulates its amino acid metabolic flux to adjust free amino acid pools, thereby contributing to osmotic balance during alkalinity exposure. In contrast, under combined saline–alkalinity stress, cholesterol metabolism emerged as the most prominently enriched pathway. Cholesterol serves not only as a structural constituent of cellular membranes [96], but also as a precursor for bile acids, steroid hormones, and vitamin D and as a substrate for many substances, including bile acids, steroid hormones, and vitamin D [97,98]. Moreover, cholesterol is integral to neuronal function, synaptogenesis, axonal plasticity, and glial cell proliferation, given its abundance in myelin sheaths [99]. Bile acids, derived from cholesterol, play a crucial role in facilitating the excretion, absorption, and transport of fats and cholesterol within the intestine and liver [100]. Notably, among the six significantly enriched pathways under saline–alkalinity stress, two were immune-related—antigen processing and presentation, and the intestinal immune network for IgA production. Collectively, these findings indicate that alkalinity and combined saline–alkalinity challenges impose more severe osmotic and disease-related stress on the intestine of *O. mossambicus* than salinity stress alone.

## 5. Conclusions

In this study, the intestinal responses of *O. mossambicus* to salinity, alkalinity, and combined saline–alkalinity challenges were comprehensively evaluated by integrating histological examination, antioxidant enzyme activity assays, and TMT-based quantitative proteomic profiling. Our analysis identified a suite of differentially expressed proteins implicated in stress adaptation and osmoregulatory processes within the intestinal tissue. To our knowledge, this is the first proteomic investigation of the tilapia intestine under these three distinct osmotic conditions, and the findings provide valuable insights into the molecular players and signaling cascades governing osmoregulation in this euryhaline species. Nevertheless, further functional characterization of the candidate proteins is required to elucidate their precise contributions to osmotic homeostasis. Given the 24-h acute exposure protocol adopted here, the present findings should be interpreted as early-phase stress responses rather than long-term osmoregulatory adaptation. Future studies employing prolonged exposure periods, larger sample sizes for the physiological assays, and functional validation of the candidate proteins (e.g., gene silencing or overexpression) are warranted to clarify their precise roles in intestinal osmoregulation, particularly under individual versus combined stress regimes.

## Data Availability

The original contributions presented in this study are included in the article/Supplementary Material. Further inquiries can be directed to the corresponding author.

## Supplementary Materials

The following supporting information can be downloaded at: https://www.mdpi.com/article/10.3390/ani16193069/s1.

## Abbreviations

ANOVA: analysis of variance; BCA: bicinchoninic acid; BP: biological process; CAT: catalase; DEPs: differentially expressed proteins; FDR: false discovery rate; FC: fold change; GO: Gene Ontology; GFR: glomerular filtration rate; GSH-Px: glutathione peroxidase; H&E: hematoxylin and eosin; KEGG: Kyoto Encyclopedia of Genes and Genomes; MDA: malondialdehyde; MS/MS: tandem mass spectrometry; NCE: normalized collision energy; NSI: nano-electrospray ionization; PRM: parallel reaction monitoring; ROS: reactive oxygen species; SD: standard deviation; SOD: superoxide dismutase; TBA: thiobarbituric acid; TEAB: tetraethylammonium bromide; TMT: tandem mass tag; UPLC: ultra-performance liquid chromatography.

## References

[1] Osmundsen T.C., Amundsen V.S., Alexander K.A., Asche F., Bailey J., Finstad B., Olsen M.S., Hernández K., Salgado H. (2020). The operationalisation of sustainability: Sustainable aquaculture production as defined by certification schemes. Glob. Environ. Change.

[2] Zaniboni-Filho E., Pedron J.d.S., Ribolli J. (2018). Opportunities and challenges for fish culture in Brazilian reservoirs: A review. Acta Limnol. Bras..

[3] Mengistu S.B., Mulder H.A., Benzie J.A.H., Komen H. (2020). A systematic literature review of the major factors causing yield gap by affecting growth, feed conversion ratio and survival in Nile tilapia (*Oreochromis niloticus*). Rev. Aquac..

[4] De Azevedo R.V., De Oliveira K.F., Flores-Lopes F., Teixeira-Lanna E.A., Takishita S.S., Tavares-Braga L.G. (2015). Responses of Nile tilapia to different levels of water salinity. Lat. Am. J. Aquat. Res..

[5] Durigon E.G., Lazzari R., Uczay J., Lopes D.L.d.A., Jerônimo G.T., Sgnaulin T., Emerenciano M.G.C. (2020). Biofloc technology (BFT): Adjusting the levels of digestible protein and digestible energy in diets of Nile tilapia juveniles raised in brackish water. Aquac. Fish..

[6] Kültz D., Bastrop R., Jürss K., Siebers D. (1992). Mitochondria-rich (MR) cells and the activities of the Na^+^K^+^-ATPase and carbonic anhydrase in the gill and opercular epithelium of *Oreochromis mossambicus* adapted to various salinities. Comp. Biochem. Physiol. Part B.

[7] Uchida K., Kaneko T., Miyazaki H., Hasegawa S., Hirano T. (2000). Excellent salinity tolerance of Mozambique tilapia (*Oreochromis mossambicus*): Elevated chloride cell activity in the branchial and opercular epithelia of the fish adapted to concentrated seawater. Zool. Sci..

[8] Kültz D., Fiol D., Valkova N., Gomez-Jimenez S., Chan S.Y., Lee J. (2007). Functional genomics and proteomics of the cellular osmotic stress response in ‘non-model’ organisms. J. Exp. Biol..

[9] Dissanayake D.C.T., Giri S.S. (2019). Milkfish aquaculture in Sri Lanka: Progress, challenges and opportunities. Aquaculture of Commercially Important Finfishes in South Asia.

[10] Durigon E.G., Almeida A.P.G., Jerônimo G.T., Baldisserotto B., Emerenciano M.G.C. (2019). Digestive enzymes and parasitology of Nile tilapia juveniles raised in brackish biofloc water and fed with different digestible protein and digestible energy levels. Aquaculture.

[11] Jiang W., Tian X., Fang Z., Li L., Dong S., Li H., Zhao K. (2019). Metabolic responses in the gills of tongue sole (*Cynoglossus semilaevis*) exposed to salinity stress using NMR-based metabolomics. Sci. Total Environ..

[12] Kültz D. (2015). Physiological mechanisms used by fish to cope with salinity stress. J. Exp. Biol..

[13] Wang J.-Q., Lui H., Po H., Fan L. (1997). Influence of salinity on food consumption, growth and energy conversion efficiency of common carp (*Cyprinus carpio*) fingerlings. Aquaculture.

[14] Xu J., Yan B., Teng Y., Lou G., Lu Z. (2010). Analysis of nutrient composition and fatty acid profiles of Japanese sea bass *Lateolabrax japonicus* (Cuvier) reared in seawater and freshwater. J. Food Compos. Anal..

[15] Saoud I.P., Kreydiyyeh S., Chalfoun A., Fakih M. (2007). Influence of salinity on survival, growth, plasma osmolality and gill Na^+^–K^+^–ATPase activity in the rabbitfish *Siganus rivulatus*. J. Exp. Mar. Biol. Ecol..

[16] Chisari M., Todaro A., Barbagallo R.N., Spagna G. (2010). Salinity effects on enzymatic browning and antioxidant capacity of fresh-cut baby Romaine lettuce (*Lactuca sativa* L. cv. Duende). Food Chem..

[17] Xu H., Ren M., Liang H., Ge X., Ji K., Huang D., Yu H., Wu L. (2022). Interactive effects of water salinity and dietary methionine levels on growth performance, whole-body composition, plasma parameters, and expression of major nutrient metabolism genes in juvenile genetically improved farmed Tilapia (*Oreochromis niloticus*). Aquaculture.

[18] Wilkie M.P., Wood C.M. (1996). The adaptations of fish to extremely alkaline environments. Comp. Biochem. Physiol. Part B.

[19] Rocha A.d.F., Ferreira T.A., Boari C.A., Pedreira M.M. (2026). Comparison Between Environmentally Friendly Recirculation Systems with Biofilter, Green Water, Bioflocs, and Partial Water Exchange in the Cultivation of Nile Tilapia. Aquacult. Stud..

[20] Zhao Y., Zhang C., Zhou H., Song L., Wang J., Zhao J. (2020). Transcriptome changes for Nile tilapia (*Oreochromis niloticus*) in response to alkalinity stress. Comp. Biochem. Physiol. Part D.

[21] Yao Z.L., Wang H., Chen L., Zhou K., Ying C.Q., Lai Q.F. (2012). Transcriptomic profiles of Japanese medaka (*Oryzias latipes*) in response to alkalinity stress. Genet. Mol. Res..

[22] Sun Y.-C., Han S.-C., Yao M.-Z., Liu H.-B., Wang Y.-M. (2020). Exploring the metabolic biomarkers and pathway changes in crucian under carbonate alkalinity exposure using high-throughput metabolomics analysis based on UPLC-ESI-QTOF-MS. RSC Adv..

[23] Yao Z., Lai Q., Hao Z., Chen L., Lin T., Zhou K., Wang H. (2015). Carbonic anhydrase 2-like and Na^+^-K^+^-ATPase α gene expression in medaka (*Oryzias latipes*) under carbonate alkalinity stress. Fish Physiol. Biochem..

[24] Martínez-Álvarez R.M., Morales A.E., Sanz A. (2005). Antioxidant defenses in fish: Biotic and abiotic factors. Rev. Fish Biol. Fish..

[25] Firdaus M.Z.A., Bello A.M., Arifin N.B., Hariati A.M., Fakhri M. (2026). Dietary Proline Enhances Physiological Adaptation and Oxidative Stress Resistance in Nile Tilapia Cultured Under Chronic Salinity Stress. Aquacult. Stud..

[26] Lesser M.P. (2006). Oxidative stress in marine environments: Biochemistry and physiological ecology. Annu. Rev. Physiol..

[27] Long X., Wu X., Zhao L., Liu J., Cheng Y. (2017). Effects of dietary supplementation with *Haematococcus pluvialis* cell powder on coloration, ovarian development and antioxidation capacity of adult female Chinese mitten crab, *Eriocheir sinensis*. Aquaculture.

[28] Long S., Dong X., Yan X., Liu H., Tan B., Zhang S., Chi S., Yang Q., Liu H., Yang Y. (2022). The effect of oxidized fish oil on antioxidant ability, histology and transcriptome in intestine of the juvenile hybrid grouper (♀ *Epinephelus fuscoguttatus* × ♂ *Epinephelus lanceolatus*). Aquac. Rep..

[29] Tran-Ngoc K.T., Schrama J.W., Le M.T.T., Nguyen T.H., Roem A.J., Verreth J.A.J. (2017). Salinity and diet composition affect digestibility and intestinal morphology in Nile tilapia (*Oreochromis niloticus*). Aquaculture.

[30] Si-Tahar M., Merlin D., Sitaraman S., Madara J.L. (2000). Constitutive and regulated secretion of secretory leukocyte proteinase inhibitor by human intestinal epithelial cells. Gastroenterology.

[31] Pitman R.S., Blumberg R.S. (2000). First line of defense: The role of the intestinal epithelium as an active component of the mucosal immune system. J. Gastroenterol..

[32] Kim H., Koedrith P., Seo Y. (2015). Ecotoxicogenomic approaches for understanding molecular mechanisms of environmental chemical toxicity using aquatic invertebrate, Daphnia model organism. Int. J. Mol. Sci..

[33] Kwan Y.H., Zhang D., Mestre N.C., Wong W.C., Wang X., Lu B., Wang C., Qian P.-Y., Sun J. (2019). Comparative proteomics on deep-sea amphipods after in situ copper exposure. Environ. Sci. Technol..

[34] Liu Z., Li Y., Pérez E., Jiang Q., Chen Q., Jiao Y., Huang Y., Yang Y., Zhao Y. (2021). Polystyrene nanoplastic induces oxidative stress, immune defense, and glycometabolism change in *Daphnia pulex*: Application of transcriptome profiling in risk assessment of nanoplastics. J. Hazard. Mater..

[35] Xu C., Li E., Liu Y., Wang X., Qin J.G., Chen L. (2017). Comparative proteome analysis of the hepatopancreas from the Pacific white shrimp *Litopenaeus vannamei* under long-term low salinity stress. J. Proteom..

[36] Chen L., Sun J., Zhang H., Au D.W.T., Lam P.K.S., Zhang W., Bajic V.B., Qiu J.-W., Qian P.-Y. (2015). Hepatic proteomic responses in marine medaka (*Oryzias melastigma*) chronically exposed to antifouling compound butenolide [5-octylfuran-2 (5H)-one] or 4, 5-dichloro-2-n-octyl-4-isothiazolin-3-one (DCOIT). Environ. Sci. Technol..

[37] Jia Y., Yin S., Li L., Li P., Liang F., Wang X., Wang X., Wang L., Su X. (2016). ITRAQ proteomic analysis of salinity acclimation proteins in the gill of tropical marbled eel (*Anguilla marmorata*). Fish Physiol. Biochem..

[38] Wang H., Wei H., Tang L., Lu J., Mu C., Wang C. (2018). A proteomics of gills approach to understanding salinity adaptation of *Scylla paramamosain*. Gene.

[39] Su H., Ma D., Fan J., Zhong Z., Tian Y., Zhu H. (2025). A TMT-Based Proteomic Analysis of Osmoregulation in the Gills of *Oreochromis mossambicus* Exposed to Three Osmotic Stresses. Int. J. Mol. Sci..

[40] Su H., Li Y., Ma D., Fan J., Zhong Z., Zhu H. (2023). Metabolism responses in the intestine of *Oreochromis mossambicus* exposed to salinity, alkalinity and salt-alkalinity stress using LC-MS/MS-based metabolomics. Comp. Biochem. Physiol. Part D.

[41] Moraes G., de Almeida L.C., Baldisserotto B., Urbinati E.C., Cyrino J.E.P. (2020). Nutrition and functional aspects of digestion in fish. Biology and Physiology of Freshwater Neotropical Fish.

[42] Wong M.K.-S., Ozaki H., Suzuki Y., Iwasaki W., Takei Y. (2014). Discovery of osmotic sensitive transcription factors in fish intestine via a transcriptomic approach. BMC Genom..

[43] Lehtonen T.K., Wong B.B.M., Kvarnemo C. (2016). Effects of salinity on nest-building behaviour in a marine fish. BMC Ecol..

[44] Dawood M.A.O., Noreldin A.E., Sewilam H. (2021). Long term salinity disrupts the hepatic function, intestinal health, and gills antioxidative status in Nile tilapia stressed with hypoxia. Ecotoxicol. Environ. Saf..

[45] Barbosa M.L., de Meneses A.-A.P.M., de Aguiar R.P.S., de Castro e Sousa J.M., de Carvalho Melo Cavalcante A.A., Maluf S.W. (2020). Oxidative stress, antioxidant defense and depressive disorders: A systematic review of biochemical and molecular markers. Neurol. Psychiatry Brain Res..

[46] Lushchak V.I. (2011). Environmentally induced oxidative stress in aquatic animals. Aquat. Toxicol..

[47] Zhou L., Zhang J., Yan M., Tang S., Wang X., Qin J.G., Chen L., Li E. (2020). Inulin alleviates hypersaline-stress induced oxidative stress and dysbiosis of gut microbiota in Nile tilapia (*Oreochromis niloticus*). Aquaculture.

[48] Birnie-Gauvin K., Costantini D., Cooke S.J., Willmore W.G. (2017). A comparative and evolutionary approach to oxidative stress in fish: A review. Fish Fish..

[49] Mohamed N.A., Saad M.F., Shukry M., El-Keredy A.M.S., Nasif O., Van Doan H., Dawood M.A.O. (2021). Physiological and ion changes of Nile tilapia (*Oreochromis niloticus*) under the effect of salinity stress. Aquac. Rep..

[50] Sun J.-L., Zhao L.-L., Liao L., Tang X.-H., Cui C., Liu Q., He K., Ma J.-D., Jin L., Yan T. (2020). Interactive effect of thermal and hypoxia on largemouth bass (*Micropterus salmoides*) gill and liver: Aggravation of oxidative stress, inhibition of immunity and promotion of cell apoptosis. Fish Shellfish Immunol..

[51] dos Santos Xavier W., Leclercq E., Carvalho P.L.P.F., Vicente I.S.T., Guimarães M.G., Rodrigues E.J.D., Milanezi R.C., Barbé F., Sartori M.M.P., Pezzato L.E. (2020). The putative effect of a SOD-rich melon pulp-concentrate on growth performance and antioxidant status of Nile tilapia (*Oreochromis niloticus*) under heat/dissolved oxygen-induced stress. Aquaculture.

[52] Ahmed R.G. (2005). Is there a balance between oxidative stress and antioxidant defense system during development?. Med. J. Islam. World Acad. Sci..

[53] Heise K., Puntarulo S., Nikinmaa M., Abele D., Pörtner H.-O. (2006). Oxidative stress during stressful heat exposure and recovery in the North Sea eelpout *Zoarces viviparus* L. J. Exp. Biol..

[54] Blier P. (2014). Fish health: An oxidative stress perspective. Fish. Aquac. J..

[55] Downs C.A., Fauth J.E., Woodley C.M. (2001). Assessing the health of grass shrimp (*Palaeomonetes pugio*) exposed to natural and anthropogenic stressors: A molecular biomarker system. Mar. Biotechnol..

[56] Xu C., Li E., Suo Y., Su Y., Lu M., Zhao Q., Qin J.G., Chen L. (2018). Histological and transcriptomic responses of two immune organs, the spleen and head kidney, in Nile tilapia (*Oreochromis niloticus*) to long-term hypersaline stress. Fish Shellfish Immunol..

[57] Saleh M., Aboueleila R., Badran M.F., Hua J., Dong Y., Lu S., Tao W.-J., Tao Y., Qiang J. (2026). Integrated histopathological, physiological, biochemical, and molecular analysis of gill responses to acute salinity stress in red tilapia (*Oreochromis* spp.). J. World Aquac. Soc..

[58] McCormick S.D., Farrell A.P., Brauner C.J. (2013). Fish Physiology: Euryhaline Fishes.

[59] Carvalho E.S.M., Gregório S.F., Canário A.V.M., Power D.M., Fuentes J. (2015). PTHrP regulates water absorption and aquaporin expression in the intestine of the marine sea bream (*Sparus aurata*, L.). Gen. Comp. Endocrinol..

[60] Martos-Sitcha J.A., Campinho M.A., Mancera J.M., Martínez-Rodríguez G., Fuentes J. (2015). Vasotocin and isotocin regulate aquaporin 1 function in the sea bream. J. Exp. Biol..

[61] Whittamore J.M. (2012). Osmoregulation and epithelial water transport: Lessons from the intestine of marine teleost fish. J. Comp. Physiol. B.

[62] Li H., Zhou Y., Ling H., Luo L., Qi D., Feng L. (2019). The effect of dietary supplementation with Clostridium butyricum on the growth performance, immunity, intestinal microbiota and disease resistance of tilapia (*Oreochromis niloticus*). PLoS ONE.

[63] Gregório S.F., Carvalho E.S.M., Encarnação S., Wilson J.M., Power D.M., Canário A.V.M., Fuentes J. (2013). Adaptation to different salinities exposes functional specialization in the intestine of the sea bream (*Sparus aurata* L.). J. Exp. Biol..

[64] Li Z., Lui E.Y., Wilson J.M., Ip Y.K., Lin Q., Lam T.J., Lam S.H. (2014). Expression of key ion transporters in the gill and esophageal-gastrointestinal tract of euryhaline Mozambique tilapia *Oreochromis mossambicus* acclimated to fresh water, seawater and hypersaline water. PLoS ONE.

[65] Wilson R.W., Wilson J.M., Grosell M. (2002). Intestinal bicarbonate secretion by marine teleost fish—Why and how?. Biochim. Biophys. Acta Biomembr..

[66] Zhou Z., Hu F., Li W., Yang X., Hallerman E., Huang Z. (2021). Effects of salinity on growth, hematological parameters, gill microstructure and transcriptome of fat greenling *Hexagrammos otakii*. Aquaculture.

[67] Zhu H., Liu Z., Gao F., Lu M., Liu Y., Su H., Ma D., Ke X., Wang M., Cao J. (2018). Characterization and expression of Na^+^/K^+^-ATPase in gills and kidneys of the Teleost fish *Oreochromis mossambicus*, *Oreochromis urolepis* hornorum and their hybrids in response to salinity challenge. Comp. Biochem. Physiol. Part A.

[68] Xia J.H., Liu P., Liu F., Lin G., Sun F., Tu R., Yue G.H. (2013). Analysis of stress-responsive transcriptome in the intestine of Asian seabass (*Lates calcarifer*) using RNA-Seq. DNA Res..

[69] Ronkin D., Seroussi E., Nitzan T., Doron-Faigenboim A., Cnaani A. (2015). Intestinal transcriptome analysis revealed differential salinity adaptation between two tilapiine species. Comp. Biochem. Physiol. Part D.

[70] Nguyen T.V., Jung H., Nguyen T.M., Hurwood D., Mather P. (2016). Evaluation of potential candidate genes involved in salinity tolerance in striped catfish (*Pangasianodon hypophthalmus*) using an RNA-Seq approach. Mar. Genom..

[71] Maier T., Güell M., Serrano L. (2009). Correlation of mRNA and protein in complex biological samples. FEBS Lett..

[72] Vogel C., Marcotte E.M. (2012). Insights into the regulation of protein abundance from proteomic and transcriptomic analyses. Nat. Rev. Genet..

[73] Raposo de Magalhães C.S.F., Cerqueira M.A.C., Schrama D., Moreira M.J.V., Boonanuntanasarn S., Rodrigues P.M.L. (2020). A Proteomics and other Omics approach in the context of farmed fish welfare and biomarker discovery. Rev. Aquac..

[74] Anderson N.L., Anderson N.G. (1998). Proteome and proteomics: New technologies, new concepts, and new words. Electrophoresis.

[75] Schauer K.L., Reddam A., Xu E.G., Wolfe L.M., Grosell M. (2018). Interrogation of the Gulf toadfish intestinal proteome response to hypersalinity exposure provides insights into osmoregulatory mechanisms and regulation of carbonate mineral precipitation. Comp. Biochem. Physiol. Part D.

[76] Khairnar S.O., Tian X., Dong S., Fang Z., Solanki B.V., Shanthanagouda H.A. (2016). Effects of the amplitude and frequency of salinity fluctuations on antioxidant responses in juvenile tongue sole, *Cynoglossus semilaevis*. Span. J. Agric. Res..

[77] Nimse S.B., Pal D. (2015). Free radicals, natural antioxidants, and their reaction mechanisms. RSC Adv..

[78] Tiwari M.K., Hägglund P.M., Møller I.M., Davies M.J., Bjerrum M.J. (2019). Copper ion/H_2_O_2_ oxidation of Cu/Zn-Superoxide dismutase: Implications for enzymatic activity and antioxidant action. Redox Biol..

[79] Wang L., Li F., Wang B., Xiang J. (2012). Structure and partial protein profiles of the peritrophic membrane (PM) from the gut of the shrimp *Litopenaeus vannamei*. Fish Shellfish Immunol..

[80] Tsuji T., Kato A., Yasuda H., Miyaji T., Luo J., Sakao Y., Ito H., Fujigaki Y., Hishida A. (2009). The dimethylthiourea-induced attenuation of cisplatin nephrotoxicity is associated with the augmented induction of heat shock proteins. Toxicol. Appl. Pharmacol..

[81] Chang A.Y.W., Chan J.Y.H., Cheng H.-L., Tsai C.-Y., Chan S.H.H. (2009). Hypoxia-inducible factor 1/heme oxygenase 1 cascade as upstream signals in the prolife role of heat shock protein 70 at rostral ventrolateral medulla during experimental brain stem death. Shock.

[82] Monkkonen T., Lewis M.T. (2017). New paradigms for the Hedgehog signaling network in mammary gland development and breast Cancer. Biochim. Biophys. Acta Rev. Cancer.

[83] Mimeault M., Batra S.K. (2010). Frequent deregulations in the hedgehog signaling network and cross-talks with the epidermal growth factor receptor pathway involved in cancer progression and targeted therapies. Pharmacol. Rev..

[84] Wu F., Zhang Y., Sun B., McMahon A.P., Wang Y. (2017). Hedgehog signaling: From basic biology to cancer therapy. Cell Chem. Biol..

[85] Abe Y., Tanaka N. (2017). Roles of the hedgehog signaling pathway in epidermal and hair follicle development, homeostasis, and cancer. J. Dev. Biol..

[86] Matz-Soja M., Rennert C., Schönefeld K., Aleithe S., Boettger J., Schmidt-Heck W., Weiss T.S., Hovhannisyan A., Zellmer S., Klöting N. (2016). Hedgehog signaling is a potent regulator of liver lipid metabolism and reveals a GLI-code associated with steatosis. eLife.

[87] Lee C., Kim J., Wang S., Sung S., Kim N., Lee H.-H., Seo Y.-S., Jung Y. (2019). Hepatoprotective effect of kombucha tea in rodent model of nonalcoholic fatty liver disease/nonalcoholic steatohepatitis. Int. J. Mol. Sci..

[88] Lee H.-J., Jo S.-B., Romer A.I., Lim H.-J., Kim M.-J., Koo S.-H., Krauss R.S., Kang J.-S. (2015). Overweight in mice and enhanced adipogenesis in vitro are associated with lack of the hedgehog coreceptor boc. Diabetes.

[89] Minh Sang H., Fotedar R. (2004). Growth, survival, haemolymph osmolality and organosomatic indices of the western king prawn (*Penaeus latisulcatus* Kishinouye, 1896) reared at different salinities. Aquaculture.

[90] Towle D.W. (1997). Molecular approaches to understanding salinity adaptation of estuarine animals. Am. Zool..

[91] Wang Y., Srinivasan K., Siddiqui M.R., George S.P., Tomar A., Khurana S. (2008). A novel role for villin in intestinal epithelial cell survival and homeostasis. J. Biol. Chem..

[92] Khurana S., George S.P. (2008). Regulation of cell structure and function by actin-binding proteins: Villin’s perspective. FEBS Lett..

[93] Nishimura H. (2017). Renin-angiotensin system in vertebrates: Phylogenetic view of structure and function. Anat. Sci. Int..

[94] Lu J.-Y., Shu M.-A., Xu B.-P., Liu G.-X., Ma Y.-Z., Guo X.-L., Liu Y. (2015). Mud crab Scylla paramamosain glutamate dehydrogenase: Molecular cloning, tissue expression and response to hyposmotic stress. Fish. Sci..

[95] Wang W.-N., Wang A.-L., Bao L., Wang J., Liu Y., Sun R.-Y. (2004). Changes of protein-bound and free amino acids in the muscle of the freshwater prawn *Macrobrachium nipponense* in different salinities. Aquaculture.

[96] Cheng Z.J., Hardy R.W. (2004). Protein and lipid sources affect cholesterol concentrations of juvenile Pacific white shrimp, *Litopenaeus vannamei* (Boone). J. Anim. Sci..

[97] Hernández P.V., Olvera-Novoa M.A., Rouse D.B. (2004). Effect of dietary cholesterol on growth and survival of juvenile redclaw crayfish *Cherax quadricarinatus* under laboratory conditions. Aquaculture.

[98] Holme M.-H., Zeng C., Southgate P.C. (2006). The effects of supplemental dietary cholesterol on growth, development and survival of mud crab, *Scylla serrata*, megalopa fed semi-purified diets. Aquaculture.

[99] Björkhem I., Meaney S. (2004). Brain cholesterol: Long secret life behind a barrier. Arterioscler. Thromb. Vasc. Biol..

[100] Sun Y., Zhang L., Jiang Z. (2024). The role of peroxisome proliferator-activated receptors in the regulation of bile acid metabolism. Basic Clin. Pharmacol. Toxicol..

